# ﻿An updated infrageneric classification of the pantropical species-rich genus *Garcinia* L. (Clusiaceae) and some insights into the systematics of New Caledonian species, based on molecular and morphological evidence

**DOI:** 10.3897/phytokeys.239.112563

**Published:** 2024-03-15

**Authors:** Myriam Gaudeul, Patrick Sweeney, Jérôme Munzinger

**Affiliations:** 1 Institut de Systématique, Evolution, Biodiversité (ISYEB), Muséum National d’Histoire Naturelle-CNRS-SU-EPHE-UA, 57 rue Cuvier, CP 39, 75231 Paris, Cedex 05, France Muséum National d’Histoire Naturelle-CNRS-SU-EPHE-UA Paris France; 2 Yale Peabody Museum, Yale University, 170 Whitney Avenue, New Haven, CT 06511, USA Yale University New Haven United States of America; 3 AMAP, University of Montpellier, IRD, INRAE, CIRAD, CNRS, Montpellier, France University of Montpellier Montpellier France

**Keywords:** Androecium, floral diversity, *Garcinia*, infrageneric classification, molecular phylogeny, morphological characters, New Caledonia, taxonomy

## Abstract

*Garcinia* L. is a pantropically distributed genus comprised of at least 250 species of shrubs and trees and has centers of diversity located in Africa/Madagascar, Australasia, and Southeast Asia. The genus is notable due to its extreme diversity of floral form, common presence in lowland tropical rainforests worldwide, and potential pharmacological value. Across its entire geographic range, *Garcinia* lacks a recent taxonomic revision, with the last genus-level taxonomic treatment of *Garcinia* conducted over 40 years ago. In order to provide an evolutionary-based framework for a revised infrageneric classification of the genus and to investigate in more detail the systematics of New Caledonian species, we conducted molecular phylogenetic analyses using DNA sequence data for the nuclear ITS region on all samples, and for three chloroplast intergenic spacers (*psbM-trnD*, *trnQ-rps16* and *rps16-trnK*) on a subset of our overall sampling. Our phylogenetic analyses are the most comprehensive to date for the genus, containing 111 biogeographically and morphologically diverse *Garcinia* species. The analyses support a broad circumscription of *Garcinia*, including several previously segregated genera (e.g. *Allanblackia*, *Clusianthemum*, *Ochrocarpos* p.p., *Pentaphalangium*, *Rheedia*, and *Tripetalum*). We recovered nine major clades falling within two major lineages, and we delimit 11 sections. We discuss each of the clades, assign them sectional names, discuss their distinguishing morphological features, compare our taxonomic treatment with the most recent sectional treatment, list representative species, note geographic distribution, and highlight some questions that deserve future investigations. We propose nine new nomenclatural combinations, four new names, and three new lectotypes. In New Caledonia (NC), a total of ten, all endemic, species are recognized and were included in our phylogenetic analyses, with several replicates per species (with the exception of *G.virgata* and *G.urceolata*, represented by a single accession each). New Caledonian species were retrieved within three separate clades, respectively including 1) *G.balansae*; 2) *G.comptonii*, *G.neglecta*, *G.urceolata*, *G.virgata*; and 3) *G.amplexicaulis*, *G.densiflora*, *G.pedicellata*, *G.puat*, *G.vieillardii*. Within NC, the phylogenies did not support the distinction between a putative undescribed species and *G.balansae*. However, it confirmed the distinction between NC species and both *G.vitiensis* (found in Fiji and Vanuatu) and *G.adinantha* (found in Fiji), suggesting that all NC species should be considered as endemics.

## ﻿Introduction

Species rich, morphologically diverse genera can benefit from the delimitation of natural infrageneric groups, which can help to facilitate future monographic work, ecological and evolutionary research, and conservation efforts (van Welzen et al. 2009; Moonlight et al. 2018; Atkins et al. 2021). *Garcinia* L. is a large genus with centers of species diversity located in Africa/Madagascar, Australasia, and Southeast Asia. The genus exhibits extreme diversity of floral morphology, particularly in the androecium and is of high ecological significance with many species forming an important component of the lower strata of lowland tropical forests worldwide. *Garcinia* is also of high economic significance since many species have edible fruits (especially *G.mangostana*) and/or possible medicinal properties (e.g., Pedraza-Chaverri et al. 2008; Espirito Santo et al. 2020).

Recent phylogenetic and biogeographic studies (e.g. Sweeney 2008; Ruhfel et al. 2011; Ruhfel et al. 2016) support a broad circumscription of *Garcinia* that justifies the inclusion of several previously segregated genera (e.g. *Ochrocarpos* Thouars p.p., *Pentaphalangium* Warb., *Rheedia* L., and *Tripetalum* K.Schum.). When broadly circumscribed, the genus contains at least 250 species (Stevens 2007) and maybe as many as ca. 400 (POWO 2023). While some of these molecular studies (Sweeney 2008) revealed major clades with suites of shared morphological characters, no recent genus-wide infrageneric classification of the genus has been attempted.

### ﻿Infrageneric taxonomy and classification of *Garcinia*

The taxonomy and systematics of *Garcinia* is made challenging due to several factors including the large number of species, dioecy, extreme floral diversity in the paleotropics (particularly in the androecium), poor preservation state of some features (e.g. fruits and flowers) on herbarium specimens, and numerous geographic sites harboring sympatric species. Several valuable efforts have been made to bring taxonomic order to the genus, at various geographic and taxonomic scales.

Previous taxonomic treatments over the past 200 years have resulted in more than 50 infrageneric taxa (Jones 1980). In the most recent worldwide taxonomic treatment of the genus and the benchmark against which more recent genus-level taxonomic work has been evaluated, Jones (1980) recognized 14 sections (Table 1). This treatment relied heavily on staminate flower and pollen morphology to classify upwards of 345 named species. Prior to Jones (1980), the most recent taxonomic treatment of the genus was that of Engler (1894, 1925), which recognized 34 sections. That work was an elaboration of Pierre (1883), who produced the first monograph of *Garcinia* (excluding *Ochrocarpos* and *Rheedia*) and used largely flower and inflorescence characters to classify 149 species into 37 sections that were organized into six groups. The only other monograph of the genus is that of Vesque (1893) who used floral morphology and leaf anatomy to classify 180 species (excluding *Rheedia*) into three subgenera and nine sections. The first major, global treatment of *Garcinia* (but narrowly circumscribed and not including the segregate genera *Discostigma*, *Ochrocarpos*, *Rheedia*, and *Xanthochymus*) was that of Planchon and Triana (1860), who used mostly floral characters to group 32 species into six sections.

**Table 1.** Sections and numbers of species recognized by Jones (1980) and their correspondence to sections and clades recognized in this study. *Allanblackia* was treated as separate from *Garcinia* by Jones (1980).

Section sensu Jones (1980)	No. spp. (sensu Jones 1980)	Clade	Section in this study
*Xanthochymus* (Roxb.) Pierre	42	1	*Xanthochymus* (Roxb.) Pierre
*Tetraphalangium* Engl.	2		
*Rheediopsis* Pierre	20	2	*Rheedia* (L.) S.W.Jones ex P.W.Sweeney
*Rheedia* (L.) S.W.Jones, *nom. inval.*	21		
*Teracentrum* Pierre	4		
*Paragarcinia* (Baillon) Vesque	10	3	*Paragarcinia* (Baillon) Vesque
*Discostigma* (Haask.) Hook.f. subsection Discostigma	53	4	*Discostigma* (Haask.) Hook.f.
*Brindonia* (Thouars) Choisy	78	5	*Brindonia* (Thouars) Choisy
*Garcinia* L.	46	6	*Garcinia* L.
*Hebradendron* (Graham) Planch. & Triana	35	7	*Hebradendron* (Graham) Planch. & Triana
*Tagmanthera* Pierre	18	8	*Tagmanthera* Pierre
*Mungotia* Pierre	9	9	*Macrostigma* Pierre
*Tripetalum* (K. Schum.) S.W.Jones, 1980, *nom. inval.*	1		
*Macrostigma* Pierre	7		
DiscostigmasubsectionDicrananthera (Pierre) S.W.Jones, *nom. inval.*	2	–	*Dicrananthera* Pierre

In addition to the above-mentioned works that are global in scope, there have been several noteworthy publications that have dealt with the genus at narrower geographic or taxonomic scales. These studies include work on species in Africa (Sosef and Dauby 2012), Australia (Cooper 2013), Brazil (Mouzinho et al. 2022), China (Li et al. 2007), Colombia (Medellín Zabala 2015), India (Maheshwari 1964; Singh 1993; Mohanan et al. 2023), and Madagascar (Sweeney and Rogers 2008; Rogers et al. 2011).

Two notable recently published works dealing with the infrageneric classification of *Garcinia* are that of Nazre et al. (2018), who provided a monograph for Garcinia and the molecular phylogenetic study of Sweeney (2008) who evaluated Jones’ (1980) classification in relation to phylogeny and morphology. Some major findings of Sweeney (2008) were that some segregated genera should be included within *Garcinia*, and while partly congruent with phylogeny, the infrageneric sectional classification of Jones (1980) needs revision.

### ﻿Taxonomy of New Caledonian *Garcinia* species

In contrast to other regions cited above, and in spite of the observed diversity within *Garcinia* in New Caledonia (NC), an archipelago that is well-known for its high overall levels of botanical diversity and endemism (Morat et al. 2012; Munzinger et al. 2023), the genus has not been recently and thoroughly studied in this territory. Only one species (*G.amplexicaulis*) was included in the phylogeny of Sweeney (2008). One species was recently described (Munzinger et al. 2021), leading to a total of ten –all presumed endemic– species, but the circumscription of some species is unclear and some questions remain about the conspecificity or, at least, the close evolutionary relationships between some non-NC and NC species that appear morphologically similar.

**Figure 1.**
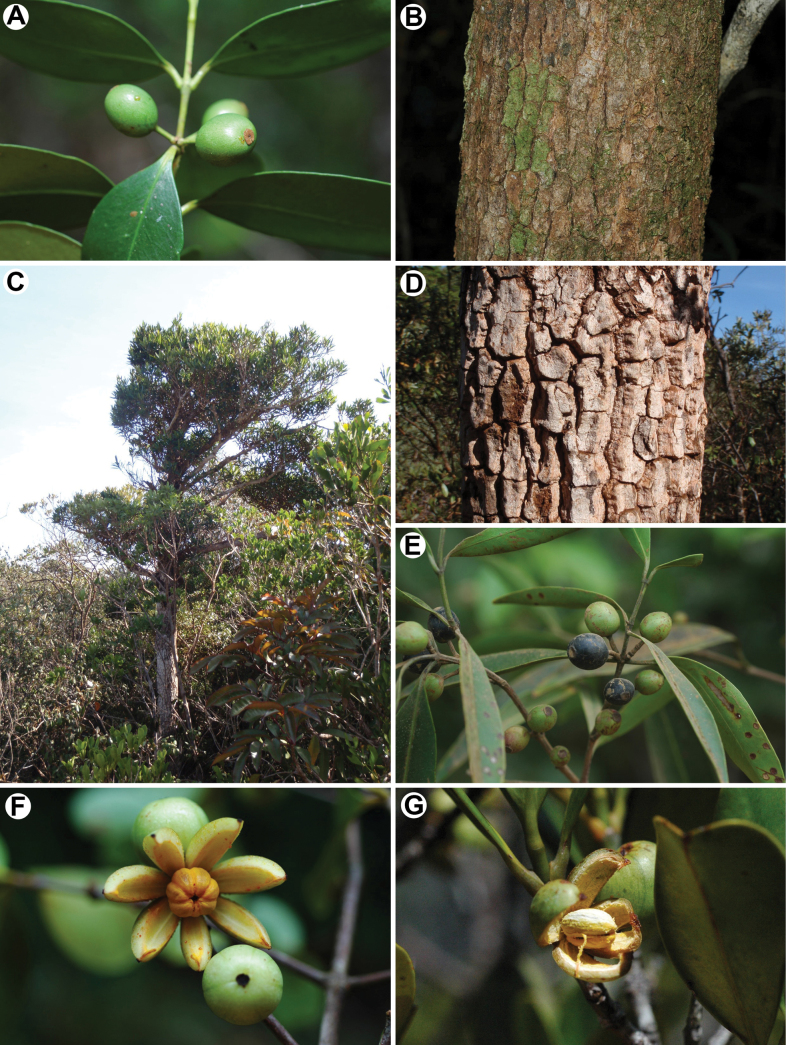
Some *Garcinia* New Caledonian species (except E from Fiji) and morphological features **A***G.balansae* (Munzinger 4916), fruiting branch **B***G.balansae* (Munzinger 4916), bark **C***G.* sp. “JT814” (Munzinger 7282), habit **D***G.* sp. “JT814” (Munzinger 7282), bark **E***G.vitiensis* (Munzinger 7377), fruiting branch **F***G.neglecta* (Munzinger 2690), fruit **G***G.comptonii* (sin voucher), fruit.

A taxon resembling *G.balansae* grows on the ultramafic massifs in the north-west of NC, but it displays linear, erect leaves and a very cracked greyish bark compared to the brownish and smoother bark of *G.balansae* (Fig. 1). This putative new taxon (*G.* sp. “JT814”) is restricted to three massifs (Boulinda, Koniambo and Tiébaghi) and should be considered as Endangered (Lowry and Munzinger 2015), but its taxonomic rank remains unresolved. In addition, the Fijian *G.vitiensis* (A. Gray) Seem. is cited in NC by Sebert and Pancher (1874), but the material of this species is then assigned by Pierre to his endemic species: *G.balansae* Pierre. Strangely, Pierre (1883: XXXVI) states “that he has never seen material of *G.vitiensis*, appearing close to *G.balansae*” (our translation). The conspecificity or non conspecificity between the two taxa remains to be tested. The presence of *G.sessilis* Seem. in NC is also mentioned at the end of the 19^th^ and in the early 20^th^ century (Sebert and Pancher 1874; Hemsley 1895), while subsequent authors considered this species as a Fijian endemic (Smith and Darwin 1974; Smith 1981) without discussion about its potential occurrence in NC. *Garciniasessilis* was later split into two species, with the description of *G.adinantha* A.C.Sm. & S.P.Darwin (Smith and Darwin 1974), but the evolutionary relationship between the New Caledonian and these two Fijian species remains unknown.

Based on an enlarged taxonomic sampling compared to Sweeney (2008), and an important sampling effort in the Pacific Ocean region and NC in particular (including several samples per morphologically delimited species), our goal is to provide an updated molecular phylogeny of the genus in order: 1) to offer a way forward on a revised infrageneric classification of *Garcinia* considering both morphological and molecular evidence; and 2) to provide insight into the systematics of NC species.

## ﻿Materials and methods

### ﻿Taxonomic sampling

This study was based on both published and newly generated sequences, leading to a total of 160 samples representing 121 species (including ten outgroups) and two putative new species (*G.* sp. “JT814” from NC and *G.* sp. Munzinger 7380 from Fiji; Suppl. material 1). Published sequences included sequences from Sweeney (2008; 57 sequences), in addition to 25 sequences downloaded from Genbank and three sequences taken from Nazre (2006). New ITS sequences were generated from both herbarium and silica-dried leaf material collected in the field. They included a total of 72 samples representing 39 species and two unidentified taxa, among which were 32 samples representing 10 species and one unidentified taxon from NC (Suppl. material 1). The sampling comprised representatives of all major *Garcinia* clades based on Sweeney (2008) and *Allanblackia*, and covered both the morphological diversity and biogeographic range of *Garcinia*. The outgroup included seven genera: *Lorostemon* Ducke, *Montrouziera* Pancher ex Planch. & Triana, *Moronobea* Aubl., *Pentadesma* Sabine and *Symphonia* L.f. from the Symphonieae tribe, and *Arawakia* L.Marinho and *Clusia* L. from the Clusieae tribe. A subset of this sampling was used to generate a fully original chloroplast DNA dataset: it comprised 67 samples representing 45 species (among which was one outgroup) and two putative new species (see above), covering all *Garcinia* clades based on Sweeney (2008) and including nine out of the ten NC*Garcinia* species.

### ﻿DNA sequencing

DNA extraction was performed with the DNeasy Plant Mini Kit (QIAGEN, Courtaboeuf, France), following the manufacturer’s protocol except for a slight modification: we added 30 µL CTAB and 30 µL proteinase K for the initial digestion, which lasted 24h at 42 °C. The nuclear ribosomal ITS region included the two transcribed intergenic spacers ITS1 and ITS2, separated by the 5.8S gene. It was sequenced using either the primers ITS4 and ITS5 (White et al. 1990) or the newly designed primers ITS4Garci (5’-CCTGACCTGGGGTCGC-3’) and ITS5Garci (5’-AACCTGCGGAAGGATCATTG-3’) that were more specific to *Garcinia* or at least to angiosperms, minimizing the risk of false positive due to fungi amplification when the amount of plant DNA was too low as a PCR template. Three chloroplast intergenic spacers were also sequenced: *psbM-trnD*, *trnQ-rps16* and *rps16-trnK*. PCR primers were psbMF and trnD^GUC^R for *psbM-trnD* (Shaw et al. 2005), trnQ^UUG^ and rps16x1 for *trnQ-rps16* (Shaw et al. 2007) and rpS16x2F2 and trnK^UUU^x1 for *rps16-trnK* (Shaw et al. 2007). All PCRs were performed in 25 µL including 1X Taq Buffer, 2.5 mM MgCl_2_, 1M betaine, 0.25 mM of each dNTP, 0.4 µM of each primer, 0.6U Taq polymerase and 1 µL template DNA. PCR conditions were: 94 °C for 5 min, followed by 40 cycles of: 94 °C 30 sec, Tm 45 sec, 72 °C 1 min, and a final extension step of 10 min at 72 °C. Tm was 48 °C for ITS and *psbM*-trnD, 44 °C for *trnQ-rps16*, and 46 °C for *rps16-trnK*. PCR products were sequenced in both directions by Eurofins (Evry, France), using the same primers as for the PCRs. Sequences were automatically aligned in MUSCLE v3.6 (Edgar 2004) before the alignments were manually revised in BioEdit v.7.2.5 (Hall 1999). Indels were coded following the simple coding method of Simmons and Ochoterena (2000) implemented in SeqState (Müller 2005). Vouchers details are listed in Suppl. material 1.

### ﻿Phylogenetic reconstructions

First, individual analyses were carried out on each DNA region. Bayesian inferences (BI) were performed using MrBayes v.3.1.2 (Ronquist et al. 2011). For each region, the best-fitting model of nucleotide substitution was identified under the Akaike information criterion in MrModelTest v.2.3 (Nylander 2004): GTR + I + Γ for the ITS region and psbM-trnD intergenic spacer, and GTR + Γ for the trnQ-rps16 and rps16-trnK intergenic spacers (using distinct models for ITS1, ITS2 and 5.8S did not make any difference in the resulting tree). For indels, we used the restriction site (binary) model, with the option lset coding = variable. Two independent but parallel analyses were conducted using flat priors, starting from random trees and consisting of four chains each. The analyses were run for 6 million generations, sampling every 1000 generations and with a 25% burn-in. Analysis of output parameters, in Tracer v.1.6 (Rambaut et al. 2014), confirmed the convergence of chains and adequate burn-in length. Post-burn-in trees were pooled and a 50% majority-rule consensus tree was computed with posterior probability (PP) estimates for all nodes. Maximum likelihood (ML) was also used to estimate phylogenetic relationships. The ML analysis was performed in raxmlGUI 1.5.1 (Silvestro and Michalak 2012; Stamatakis 2014), using the same partitions and models of nucleotide evolution as for the BI. We performed 1000 rapid bootstrap (BS) replicates and searched for the best-scoring ML tree. The topologies inferred by BI analyses from each chloroplast marker were visually compared to identify potential incongruence among them (Suppl. materials 2–4). Since no major incongruence was highly supported, the three chloroplast sequences for each sample were then combined to maximise the number of characters analysed in the BI and ML analyses. Also, a BI analysis was performed by merging the nuclear and chloroplast datasets on the reduced sampling (Suppl. material 5).

## ﻿Results

### ﻿Large-scale infrageneric phylogeny

**Figure 2.**
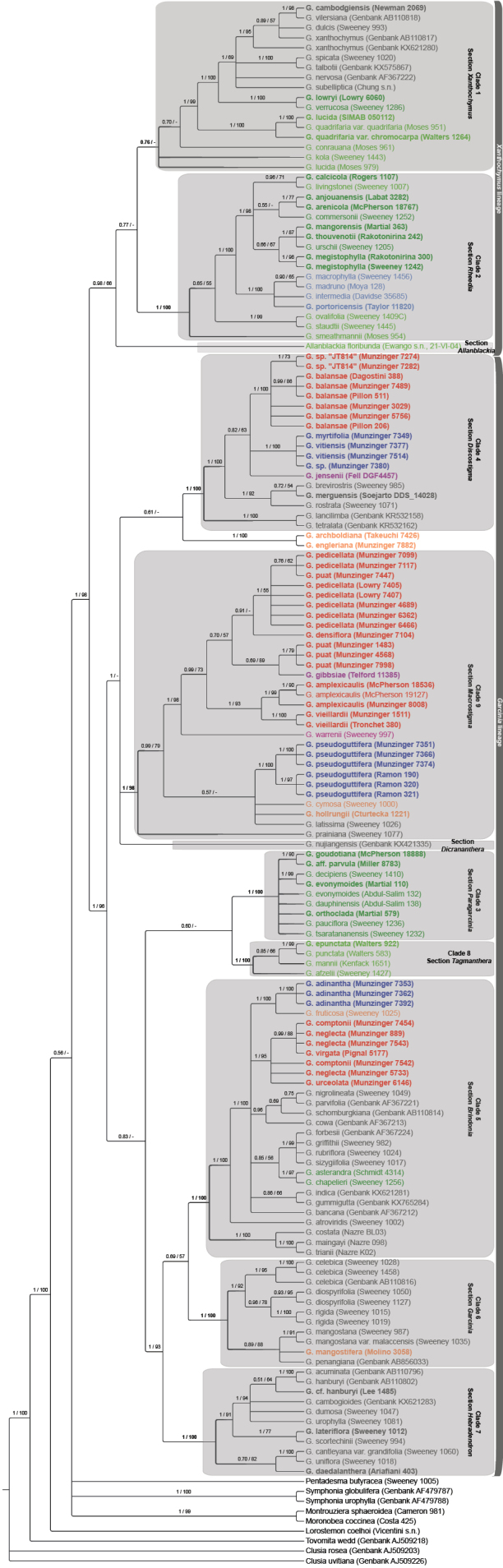
Molecular phylogeny of *Garcinia* L. based on ITS sequences and Bayesian inference. Posterior probabilities (PP) and bootstrap support values (BS), obtained respectively by the Bayesian inference and Maximum Likelihood (ML) analysis, are indicated at each node of the cladogram. Nodes were collapsed when PP < 0.50. The lineages/sections discussed in the text are highlighted, and species names appear in colors depending on their native distribution areas: light blue, Central and South America; light green, Tropical Africa; dark green, Madagascar and Western Indian Ocean islands; grey, Southeast Asia; purple, Australia; orange, New Guinea; red, New Caledonia; dark blue, Southwest Pacific islands. Distribution information was taken from the Plants of the World Online website (POWO 2023; also see the table of vouchers). A few species occur in several regions, and the color of the main (largest) geographic region was used. Accessions in bold were newly sequenced in this study.

**Figure 3.**
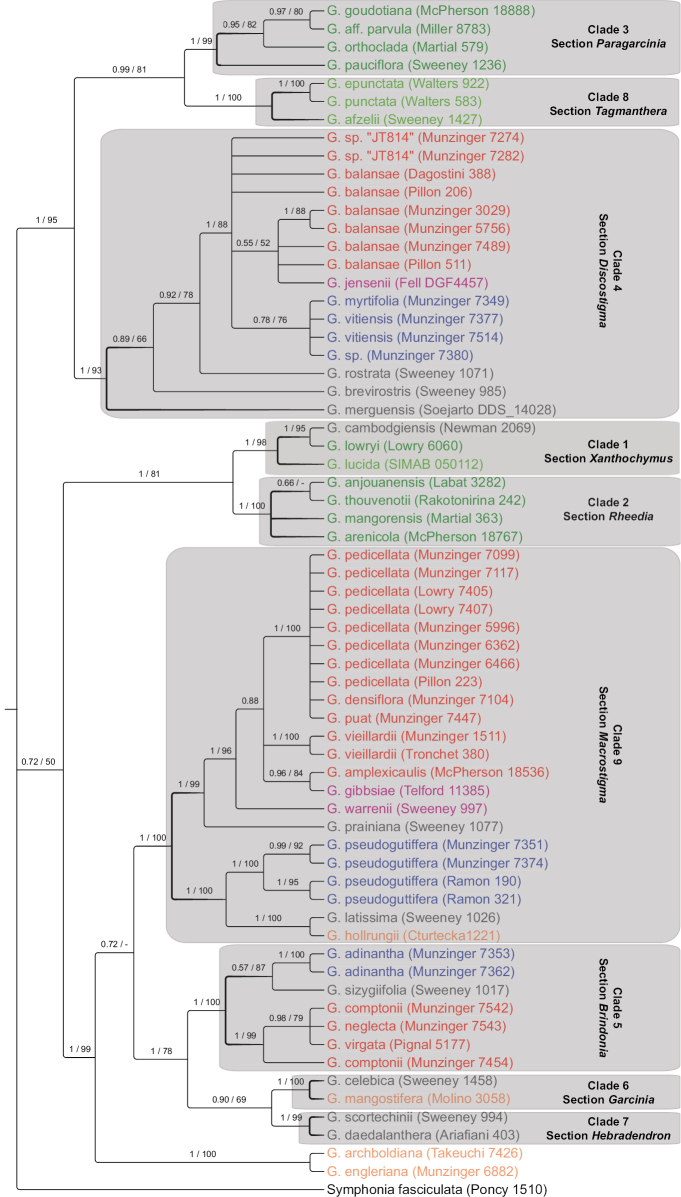
Molecular phylogeny of *Garcinia* L. based on a combined chloroplast DNA dataset and Bayesian inference. Posterior probabilities (PP) and bootstrap support values (BS), obtained respectively by the Bayesian inference and Maximum Likelihood (ML) analysis, are indicated at each node of the cladogram. Nodes were collapsed when PP < 0.50. The lineages/sections discussed in the text are highlighted, and species names appear in colors depending on their native distribution areas: light green, Tropical Africa; dark green, Madagascar and Western Indian Ocean islands; grey, Southeast Asia; purple, Australia; orange, New Guinea; red, New Caledonia; dark blue, Southwest Pacific islands. Distribution information was taken from the Plants of the World Online website (POWO 2023; also see the table of vouchers). A few species occur in several regions, and the color of the main (largest) region was used. All accessions were newly sequenced in this study.

The ITS alignment was 773 base pairs (bp) long and 91 indels were coded, whereas the cpDNA alignment was 2484 bp long (795 bp for psbM-trnD, 674 bp for trnQ-rps16 and 1015 bp for rps16-trnK) and 167 indels were coded. Only minor differences were identified among trees using BI and ML, and no conflict was supported. Because both resolution and support were higher using BI, we chose to present the resulting BI topologies, on which we also indicated the support values obtained from the ML analyses (Figs 2, 3).

Based on ITS, we recovered mostly the same two major lineages and nine clades as Sweeney (2008). The allied genera *Allanblackia*, *Ochrocarpos*, *Pentaphalangium*, *Rheedia*, and *Tripetalum* were again retrieved within *Garcinia*. We retrieved a clade containing clade 1 and clade 2, which corresponds to Lineage A in Sweeney (2008). Its support was PP = 0.77 (vs. 0.99 in Sweeney 2008) and it was not retrieved in ML. Nevertheless, the clade consisting of lineage A of Sweeney (2008) and *Allanblackiafloribunda* was more strongly supported (PP = 0.98, BS = 66). Clade 1 was not highly supported in BI (PP = 0.76 vs. 0.98 in Sweeney 2008) and not fully retrieved in ML. This was due to three species: *G.conrauana*, *G.kola* and *G.lucida*. The support of clade 1 excluding these three species reached PP = 1 and BS = 99. Compared to Sweeney (2008), nine new species were assigned to this clade (five downloaded from Genbank, three newly sequenced and *G.conrauana*). Clade 2 was strongly supported (PP = 1, BS = 100). Seven newly sequenced species were assigned to this clade. A clade corresponding to Lineage B of Sweeney (2008) consisted of clades 3 to 9 and three additional species, *G.engleriana*, *G.archboldiana* and *G.nujiangensis*, which were not included in any major clade. The Lineage B clade was highly supported (PP = 1, BS = 96) and all major subclades also received strong support (all PP = 1 and all BS = 100 except for clade 9, BS = 98). They included one (in clade 8) to 15 (in clade 5) additional species each compared to Sweeney (2008). Relationships between clades remained largely unresolved. No grouping was supported except the one including clades 5, 6 and 7 (PP = 1, BS = 93), as was observed by Sweeney (2008).

Based on the combined chloroplast dataset, the same nine clades were retrieved with high support (all PP = 1 and BS from 93 to 100). The only allied genus included in the analysis was *Pentaphalangium*, which was retrieved within *Garcinia* in agreement with the ITS phylogeny. *Garciniaarchboldiana* and *G.engleriana* were again sister species, and not included in any major clade. Clades 1 and 2 grouped together (PP = 1, BS = 81), as did clades 3 and 8 (PP = 0.99, BS = 81), which were sister to clade 4 (PP = 1, BS = 95). Clades 5, 6 and 7 grouped together (PP = 1, BS = 78) and this clade grouped with clade 9 and *G.archboldiana* and *G.engleriana* (PP = 1, BS = 99).

### ﻿Focus on the New Caledonian species

New Caledonian species were retrieved within three distinct clades: *G.balansae* and *G.* sp. “JT814” were recovered in clade 4; *G.comptonii*, *G.neglecta*, *G.urceolata* and *G.virgata* were placed in clade 5, within which they formed a highly supported subclade (PP = 1 and BS = 95 in the more densely sampled ITS phylogeny); and *G.amplexicaulis*, *G.densiflora*, *G.pedicellata*, *G.puat* and *G.vieillardii* were recovered in clade 9, grouped in a subclade that also included the Australian *G.gibbsiae* (PP = 0.99, BS = 73 in the ITS phylogeny). Together with *G.warrenii*, they formed a strongly supported clade (PP = 1, BS = 98). Within clade 4, the two accessions of *G.* sp. “JT814” formed a subclade based on ITS, but were scattered among the *G.balansae* accessions based on cpDNA. Similarly, the NC species grouped within clades 5 and 9 did not appear reciprocally monophyletic, neither in the ITS nor in the cpDNA trees (combined analyses, including both ITS and cpDNA data, did not allow a better discrimination). The only exceptions were *G.vieillardii* (two samples from the same locality; PP = 1 and BS = 100 both based on ITS and on cpDNA) and *G.amplexicaulis* (three samples, PP = 1 and BS = 99 based on ITS; only one sample in cpDNA). *Garciniavieillardii* and *G.amplexicaulis* were also sister species with high support based on ITS (PP = 1 and BS = 93), but not based on cpDNA. Both the ITS and (to a lesser extent) the cpDNA trees show that *G.vitiensis* from Fiji, although belonging to clade 4, was distinct from any of the NC taxa and more related to others Fijian species and to the *G.vitiensis* accession from Vanuatu. In clade 5, *Garciniaadinantha* was also closer to *G.fruticosa* (based on the ITS phylogeny, which was more densely sampled) than to NC species.

## ﻿Discussion

The previous most comprehensive phylogeny for *Garcinia* (*sensu lato*) included 53 species (Sweeney 2008), and the present study increases this number to 111 and includes a biogeographically and morphologically diverse set of species. This more dense and diverse sampling allows for a robust evaluation of the infrageneric classification of the genus, in relation to the morphology-based sections delineated by Jones (1980). Additionally, the inclusion of 32 samples representing the ten species endemic to New Caledonia provides an opportunity to explore in more depth the evolution and taxonomy of New Caledonian *Garcinia*.

### ﻿Taxonomy: proposal for an updated infrageneric classification of Garcinia

This study, like others (e.g. Sweeney 2008; Ruhfel et al. 2011; Ruhfel et al. 2016), supports a broad circumscription of *Garcinia* that justifies the inclusion of several previously segregated genera (e.g. *Allanblackia*, *Ochrocarpos* p.p., *Pentaphalangium*, *Rheedia*, and *Tripetalum*). The phylogenetic analyses in this paper and in Sweeney (2008) reveal nine major clades falling within two major lineages and provide a framework for a revised sectional classification of the genus. Seven of the major clades roughly correspond to sections recognized by Jones (1980; Table 1. Of the two remaining major clades, one (clade 2) is a morphologically cohesive group that is comprised chiefly of species that Jones (1980) placed into sections *Rheedia*, *Rheediopsis*, and *Teracentrum*. The other remaining major clade (clade 9) contains primarily species that were placed by Jones (1980) into sections *Macrostigma*, *Mungotia*, and *Tripetalum*. Below we discuss each of these clades, providing their distinguishing characteristics and assigning them sectional names, with the aim of laying the foundation for a phylogenetically informed infrageneric classification of *Garcinia*. The distinguishing sectional characters below are taken from the literature and from examination of physical and digitized herbarium specimens (A, BO, CNS, G, GH, K, KEP, KLU, L, MEL, MO, NY, P, PE, SCA, SING, TAN, TEF, US, YU) and plants in the field. We refer readers to Jones (1980) for a detailed account of previous infrageneric classifications of *Garcinia*, along with sectional synonymy. Informed by an examination of specimens, protologues, and Jones’ (1980) sectional assignments, we assign accepted species to sections. In cases where protologue descriptions or specimens were insufficient for determining sectional assignment, species were unplaced.

### ﻿Taxonomic account

**Genus *Garcinia* L. Sp. Pl. 1: 443 (1753).**

**Type.***Garciniamangostana* L., Sp. Pl. 1: 443 (1753).

**Synonyms.***Rheedia* L., Sp. Pl. 2: 1193 (1753). Type. *Rheedialateriflora* L. [=*Garciniahumilis* (Vahl.) C.D.Adams, Phytologia 20(5): 312 (1970); non *Garcinialateriflora* Blume, Bijdr. Fl. Ned. Ind. 5: 214 (1825)].

*Cambogia* L., Gen. Pl., ed. 5: 225 (1754). Type. *Cambogiagummi-gutta* L., Gen. Pl., ed. 5: 225 (1754) [≡*Garciniagummi-gutta* (L.) N.Robson, Brittonia 20: 103 (1968)].

*Coddampuli* Adans., Fam. Pl. (Adanson) 2: 445 (1763), *nom. illeg. superfl.* Type: *Cambogiagummi-gutta* L., Gen. Pl., ed. 5: 225 (1754) [≡*Garciniagummi-gutta* (L.) N.Robson, Brittonia 20: 103 (1968)].

*Mangostan* Garcin ex Adans., Fam. Pl. (Adanson) 2: 445 (1763), *nom. illeg. superfl.* Type. *Garciniamangostana* L., Sp. Pl. 1: 443 (1753).

*Biwaldia* Scop., Intr. Hist. Nat. 232 (1777), *nom. illeg. superfl.* Type. *Garciniamangostana* L., Sp. Pl. 1: 443 (1753).

*Stalagmitis* Murray, Commentat. Soc. Regiae Sci. Gott. 9: 173 (1789). Type. *Stalagmitiscambogioides* Murray, Commentat. Soc. Regiae Sci. Gott. 9: 173 (1789) [≡*Garciniacambogioides* (Murray) Headland, Man. Mater. Med. Therap. [Royle], ed. 3. 339 (1856)].

*Oxycarpus* Lour., Fl. Cochinch. 2: 647 (1790). Type. *Oxycarpuscochinchinensis* Lour., Fl. Cochinch. 2: 648 (1790) [≡*Garciniacochinchinensis* (Lour.) Choisy, Prodr. [A. P. de Candolle] 1: 561 (1824)].

*Mangostana* Rumph. ex Gaertn., Fruct. Sem. Pl. ii. 105. t. 105. (1791), *nom. illeg. superfl.* Type. *Garciniamangostana* L., Sp. Pl. 1: 443 (1753).

*Verticillaria* Ruiz & Pav., Fl. Peruv. Prodr. 81, t. 15 (1794). Type. *Verticillariaacuminata* Ruiz & Pav., Syst. Veg. Fl. Peruv. Chil. 1: 140 (1798) [=*Garciniamadruno* (Kunth) Hammel, Ann. Missouri Bot. Gard. 76: 928 (1989)].

*Ochrocarpos* Noronha ex Thouars, Gen. Nov. Madagasc. 15 (1805). Type. *Ochrocarposmadagascarensis* Choisy, Prodr. [A. P. de Candolle] 1: 560 (1824) [non *Ochrocarposmadagascariensis* Planchon & Triana, Ann. Sci. Nat. Bot., sér. 4, 14: 364 (1860)], see Sprague (1934) and Sweeney and Rogers (2008) for discussions about original material of *Ochrocarposmadagascarensis* and the type species of *Ochrocarpos*.

*Xanthochymus* Roxb., Pl. Coromandel 2(4): 51, t. 196 (1805). Type. *Xanthochymuspictorius* Roxb. [≡*Garciniaxanthochymus* Hook.f. ex T. Anderson Fl. Brit. India [J. D. Hooker] 1(2): 269 (1874)].

*Brindonia* Thouars, Dict. Sci. Nat. [F. Cuvier] 5: 339 (1806). Type. *Brindoniaoxycarpa* Thouars, Hist. Veg. Isles Austr. Afr. ed. 2 t. 27 (1805) [≡*Garciniaoxycarpa* (Thouars) P.W.Sweeney comb. nov.]. See commentary under Section Brindonia for details about the status of *B.oxycarpa*.

*Chloromyron* Pers., Syn. Pl. [Persoon] 2(1): 73 (1806). Type. *Chloromyronverticillatum* Pers., Syn. Pl. [Persoon] 2(1): 73 (1806) [≡*Verticillariaacuminata* Ruiz & Pav., Syst. Veg. Fl. Peruv. Chil. 1: 140 (1798); =*Garciniamadruno* (Kunth) Hammel, Ann. Missouri Bot. Gard. 76: 928 (1989)].

*Hebradendron* Graham, Companion Bot. Mag. 2: 199 (1837), *nom. illeg. superfl.* (Art. 58.1). Type. *Stalagmitiscambogioides* Murray, Commentat. Soc. Regiae Sci. Gott. Ix. 1787-88 (1789) 173. [≡*Garciniacambogioides* (Murray) Headland, Man. Mater. Med. Therap. [Royle], ed. 3. 339 (1856); ≡*Hebradendroncambogioides* (Murray) Graham, Companion Bot. Mag. 2: 199, t. 27 (1837)].

*Discostigma* Hassk., Flora 25(2, Beibl.): 33 (1842). Type. *Discostigmarostratum* Hassk., Flora 25(2, Beibl.): 33 (1842) [≡*Garciniarostrata* (Hassk.) Miq., Ann. Mus. Bot. Lugduno-Batavi 1(7): 209 (1864)].

*Terpnophyllum* Thwaites, Hooker’s J. Bot. Kew Gard. Misc. 6: 70, t. 2 C (1854). Type. *Terpnophyllumzeylanicum* Thwaites, Hooker’s J. Bot. Kew Gard. Misc. 6: 70, t. 2. F. 1 (1854) [≡*Garciniaterpnophylla* Thwaites, Enum. Pl. Zeyl. [Thwaites] 406 (1864)].

*Rhinostigma* Miq., Fl. Ned. Ind., Eerste Bijv. Pt. 3: 495 (1861). Type. *Rhinostigmaparvifolium* Miq., Fl. Ned. Ind., Eerste Bijv. Pt. 3: 495 (1861) (lectotype, designated here) [≡*Garciniaparvifolia* (Miq.) Miq., Ann. Mus. Bot. Lugduno-Batavi 1(7): 208 (1864)].

*Clusianthemum* Vieill., Bull. Soc. Linn. Normandie 9: 338 (1865). Type. *Clusianthemumpedicellatum* Vieill., Bull. Soc. Linn. Normandie 9: 339 (1865).

*Allanblackia* Oliv., Gen. Pl. [Benth. & Hook.f.] 1(3): 980 (1867), J. Linn. Soc., Bot. 10: 43 (1867). Type. *Allanblackiafloribunda* Oliv., J. Linn. Soc., Bot. 10: 43 (1867).

*Pentaphalangium* Warb., Bot. Jahrb. Syst. 13(3–4): 382 (1891). Type. *Pentaphalangiumcrassinerve* Warb., Bot. Jahrb. Syst. 13(3–4): 382 (1891) [≡*Garciniacrassinervis* (Warb.) Kosterm., Ceylon J. Sci., Biol. Sci. 12(1): 68 (1976)].

*Tripetalum* K.Schum., Fl. Kais. Wilh. Land [K.M. Schumann & M.U. Hollrung] 51 (1889). Type. *Tripetalumcymosum* K.Schum., Fl. Kais. Wilh. Land [K.M. Schumann & M.U. Hollrung] 51 (1889) [≡*Garciniacymosa* (K.Schum.) I.M.Turner & P.F.Stevens, Gard. Bull. Singapore 51(2): 176 (1999)].

*Tsimatimia* Jum. & H.Perrier, Ann. Sci. Nat., Bot. sér. 9, 11: 256 (1910). Type. *Tsimatimiapedicellata* Jum. & H.Perrier, Ann. Sci. Nat., Bot. sér. 9, 11: 265 (1910) (lectotype, designated here) [≡*Garciniatsimatimia* P.W.Sweeney & Z.S.Rogers, Novon 18(4): 535 (2008)].

*Septogarcinia* Kosterm., Reinwardtia 6: 167 (1962). Type. *Septogarciniasumbawaensis* Kosterm., Reinwardtia 6: 167 (1962) [≡*Garciniaseptogarcinia* I.M. Turner & L.V.S. Jenn; non *Garciniasumbawensis* Lauterb., Bot. Jahrb. Syst. 58(1): 26 (1922)].

#### *Xanthochymus* Lineage

***Leaves*** with prismatic crystals in the mesophyll (this character is unstudied in *Allanblackia*) (﻿Vesque 1889, 1893). ***Flowers*** with nectariferous antesepalous appendages or intrastaminal rings and disks (sometimes manifested as lobe-like antesepalous appendages that alternate with staminodes) (Sweeney 2008, 2010; Mathew et al. 2009, Fig. 1). Staminate flowers lacking pistillodes (rarely present and then rudimentary) and anthers with globose to widely elliptic thecae.

The *Xanthochymus* lineage is comprised of Lineage A in Sweeney (2008) and *Allanblackia*. It contains three sections as circumscribed below.

##### ﻿1. Garcinia section Xanthochymus

Taxon classificationPlantaeMalpighialesClusiaceae

(Roxb.) Pierre, Fl. Forest. Cochinch. 1, Fasc. 5, 3 (1883). Clade 1

65BDD8DA-C4D9-5182-8543-18A00D11B987

Figs 2 , 3

###### Basionym.

*Xanthochymus* Roxb., Pl. Coromandel 2(4): 51, t. 196 (1805).

###### Type.

*Xanthochymuspictorius* Roxb. [≡*Garciniaxanthochymus* Hook.f. ex T. Anderson Fl. Brit. India [J. D. Hooker] 1(2): 269 (1874)].

###### Distinguishing sectional characters.

***Flowers*** usually five-merous (rarely four-merous). Staminate flowers with stamens united into fascicles with filaments united for at least ½ (usually considerably more) of their length. ﻿Pollen five- to seven-colporate (Jones 1980). Ovaries with five (rarely four) locules and a single ovule per locule, placentation axile. Afrotropics, Indomalaya, and tropical Australasia.

This section largely corresponds Xanthochymus sensu Jones (1980); however, based on molecular data [this study and Sweeney (2008)] and morphology, we exclude *G.hollrungii* Lauterb. and *G.prainiana* King (and the closely allied *G.phuongmaiensis* V.S.Dang, H.Toyama & D.L.A.Tuan). We also include here *G.conrauana* Engl. and *G.giadidii* De Wild. [=*G.kola* Heckel] – the only two species that Jones (1980) placed into Tetraphalangium Engl., Bot. Jahrb. Syst. 40(4): 562 (1908), Type. *Garciniaconrauana* Engl.

###### Species.

*Garciniacambodgiensis* Vesque; *G.capuronii* Z.S.Rogers & P.W.Sweeney; *G.conrauana* Engl.; *G.densivenia* Engl.; *G.dulcis* (Roxb.) Kurz; *G.gamblei* Shameer, T.Sabu & N.Mohanan; *G.gerrardii* Harv. ex Sim; *G.kola* Heckel; *G.letestui* Pellegr.; *G.longifolia* Blume; *G.lowryi* Z.S.Rogers & P.W.Sweeney; *G.lucida* Vesque; *G.nervosa* (Miq.) Miq.; *G.petiolaris* Pierre; *G.pushpangadaniana* T.Sabu, N.Mohanan, Krishnaraj & Shareef; *G.quadrifaria* (Oliv.) Baill. ex Pierre; *G.spectabilis* Pierre; *G.spicata* (Wight & Arn.) Hook.f.; *G.subelliptica* Merr.; *G.talbotii* Raizada ex Santapau; *G.thwaitesii* Pierre; *G.verrucosa* Jum. & H.Perrier; *G.vidalii* Merr.; *G.vilersiana* Pierre; *G.volkensii* Engl.; *G.vriesiana* Pierre; *G.warburgiana* A.C.Sm.; *G.xanthochymus* Hook.f. ex T.Anderson.

##### ﻿2. Garcinia section Rheedia

Taxon classificationPlantaeMalpighialesClusiaceae

(L.) S.W.Jones ex P.W.Sweeney, comb. & stat. nov. Clade 2

54D78E09-941E-5793-903B-7FDC92784112

urn:lsid:ipni.org:names:77338399-1

Figs 2 , 3

###### Basionym.

*Rheedia* L., Sp. Pl. 2: 1193 (1753).

###### Type.

*Rheedialateriflora* L. [=*Garciniahumilis* (Vahl.) C.D.Adams, Phytologia 20(5): 312 (1970); non *Garcinialateriflora* Blume, Bijdr. Fl. Ned. Ind. 5: 214 (1825)].

###### Distinguishing sectional characters.

***Flowers*** usually with four petals (sepal number varies from two to five). Staminate flowers with stamens free or united into fascicles with filaments united up to ½ (rarely up to 2/3) of their length. Pollen tri-colporate with long ectoaperatures and endocolpi (Jones 1980). Ovaries with two to four locules and a single ovule per locule, placentation axile. Vesque (1893:288) noted that the leaves of species included in this section (i.e., species placed in the genus *Rheedia* and GarciniasubgenusRheediopsis in his monograph) have sunken stomata with raised papilla-like protuberances arising from the accessory cells and partially covering the stomatal opening. Neotropics and Afrotropics.

This section includes species placed by Jones (1980) into sections *Rheedia* (L.) S.W.Jones *nom. inval.* (Art. 30.9, Turland et al. 2018); *Rheediopsis* Pierre, Fl. Forest. Cochinch. 1, Fasc. 5, 2 (1883), Type. *G.smeathmannii* (Planch. & Triana) N.Robson ex Spirl. (lectotype, designated here); and *Teracentrum* Pierre, Fl. Forest. Cochinch. 1, Fasc. 5, 1 (1883), Type. *G.livingstonei* T. Anderson. This section includes species that were formerly placed into the genus *Rheedia* L.

###### Species.

*Garciniaalbuquerquei* (M.E.Berg) Bittrich; *G.ambrensis* (H.Perrier) P.W.Sweeney & Z.S.Rogers; *G.anjouanensis* (H.Perrier) P.W.Sweeney & Z.S.Rogers; *G.aphanophlebia* Baker; *G.apostoloi* Mouzinho; *G.arenicola* (Jum. & H.Perrier) P.W.Sweeney & Z.S.Rogers; *G.aristata* (Griseb.) Borhidi; *G.bakeriana* (Urb.) Borhidi; *G.barkeriana* (Urb. & Ekman) Alain; *G.benthamiana* (Planch. & Triana) Pipoly; *G.brasiliensis* Mart.; *G.calcicola* (Jum. & H.Perrier) P.W.Sweeney & Z.S.Rogers; *G.cincta* (Urb.) Borhidi; *G.clarensis* Borhidi; *G.commersonii* (Planch. & Triana) Vesque; *G.cubensis* (Borhidi) Borhidi; *G.dalleizettei* (H.Perrier) P.W.Sweeney & Z.S.Rogers; *G.decussata* C.D.Adams; *G.floribunda* Miq.; *G.fluviatilis* Mouzinho & L.Marinho; *G.gabonensis* Sosef & Dauby; *G.gardneriana* (Planch. & Triana) Zappi; G.×guacopary (S.Moore) M.Nee; *G.hessii* (Britton) Alain; *G.humilis* (Vahl) C.D.Adams; *G.intermedia* (Pittier) Hammel; *G.kingaensis* Engl.; *G.leptophylla* Bittrich; *G.livingstonei* T.Anderson; *G.macrophylla* Mart.; *G.madruno* (Kunth) Hammel; *G.magnifolia* (Pittier) Hammel; *G.magnophylla* (Cuatrec.) Hammel; *G.mangorensis* (R.Vig. & Humbert) P.W.Sweeney & Z.S.Rogers; *G.martinii* (Maguire) Govaerts; *G.megistophylla* P.W.Sweeney & Z.S.Rogers; *G.moaensis* (Bisse) Borhidi; *G.obliqua* Sosef & Dauby; *G.ophiticola* (Borhidi) Borhidi; *G.ovalifolia* Oliv.; *G.pachyclada* N.Robson; *G.parviflora* Benth.; *G.pervillei* (Planch. & Triana) Vesque; *G.polyneura* (Urb.) Borhidi; *G.portoricensis* (Urb.) Alain; *G.pulvinata* (Planch. & Triana) Hammel; *G.pungens* Borhidi; *G.revoluta* (Urb.) Borhidi; *G.robsoniana* Bamps; *G.ruscifolia* (Griseb.) Borhidi; *G.semseii* Verdc.; *G.serpentini* Borhidi; *G.smeathmannii* (Planch. & Triana) Oliv.; *G.spruceana* (Engl.) Mouzinho; *G.staudtii* Engl.; *G.thouvenotii* (H.Perrier) P.W.Sweeney & Z.S.Rogers; *G.tsimatimia* P.W.Sweeney & Z.S.Rogers; *G.urschii* (H.Perrier) P.W.Sweeney & Z.S.Rogers; *G.verticillata* Alain.

##### ﻿3. Garcinia section Allanblackia

Taxon classificationPlantaeMalpighialesClusiaceae

(Oliv.) P.W. Sweeney, comb. & stat. nov.

3CDD7AE2-6129-581C-91E7-5E93D73AE3FE

urn:lsid:ipni.org:names:77338400-1

###### Basionym.

*Allanblackia* Oliv., Gen. Pl. [Benth. & Hook.f.] 1(3): 980 (1867), J. Linn. Soc., Bot. 10: 43 (1867).

###### Type.

*Allanblackiafloribunda* Oliv., J. Linn. Soc., Bot. 10: 43 (1867) [≡*Garciniaoleosperma* P.W. Sweeney, nom. nov.; non *Garciniafloribunda* Miq., Stip. Surin. Sel. 39, non *Garciniafloribunda* Mast. ex Vesque, Monogr. Phan. [A.DC. & C.DC.] 8: 488 (1893)]

###### Distinguishing sectional characters.

***Flowers*** five-merous. Staminate flowers with stamens united into five phalanges, anthers subsessile, two-thecous. Pollen 4-colporate (Jones 1980). Ovaries incompletely five-locular containing multiple ovules per carpel/locule, placentation parietal. Afrotropics.

###### Note.

There are nine currently accepted species in the genus *Allanblackia* Oliv., all native to Africa (Bamps 1969; Stevens 2007; POWO 2023). Here we create the Allanblackia (Oliv.) P.W. Sweeney for these species when they are treated as *Garcinia* and below provide names for them in *Garcinia*.

**Species**:

##### ﻿ Garcinia guineensis

Taxon classificationPlantaeMalpighialesClusiaceae

P.W.Sweeney nom. nov.

CCE24C7F-8A8D-5FC8-8479-DDD2CC97F269

urn:lsid:ipni.org:names:77338401-1

Allanblackia parviflora A.Chev., Vég. Ut. Afr. Trop. Franç. 5: 163 (1909). Type. Côte d’Ivoire: Alépé, *Chevalier 16239*.

###### Note.

A replacement name (“nom. nov.”), *Garciniaguineensis*, is created here for *Allanblackiaparviflora*, because the epithet *parviflora* was used previously in *Garcinia* for a different species. The epithet *guineensis* is chosen to reflect the distribution of this species in the Upper Guinean Forest region of West Africa.

##### ﻿ Garcinia kisonghi

Taxon classificationPlantaeMalpighialesClusiaceae

(Vermoesen) P.W.Sweeney comb. nov.

2248C298-79EA-5B68-878A-1748CDF7AC89

urn:lsid:ipni.org:names:77338402-1

Allanblackia kisonghi Vermoesen, Man. Ess. Forest. Congo: 11 (1923). Type. Democratic Republic of the Congo: Mpse, *Van Naemen in Gillet s.n.*

##### ﻿ Garcinia kimbiliensis

Taxon classificationPlantaeMalpighialesClusiaceae

(Spirlet) P.W.Sweeney comb. nov.

AA9A771D-AAFF-5D82-970B-4E91AFE7B21A

urn:lsid:ipni.org:names:77338403-1

Allanblackia kimbiliensis Spirlet, Bull. Jard. Bot. État Bruxelles 29: 357 (1959). Type. Democratic Republic of the Congo: Kimbili, *Michelson 766*.

##### ﻿ Garcinia marienii

Taxon classificationPlantaeMalpighialesClusiaceae

(Staner) P.W.Sweeney comb. nov.

ECCBBADA-D054-50E5-B4B6-C624EB522C2A

urn:lsid:ipni.org:names:77338404-1

Allanblackia marienii Staner, Bull. Jard. Bot. État Bruxelles 13: 110 (1934). Type. Democratic Republic of the Congo: Haute Nsele, *De Groof s.n.*

##### ﻿ Garcinia ngouniensis

Taxon classificationPlantaeMalpighialesClusiaceae

P.W.Sweeney nom. nov.

7BB4714D-EEA2-5045-A5AA-22DFA3EC3AD5

urn:lsid:ipni.org:names:77338405-1

Allanblackia gabonensis (Pellegr.) Bamps, Bull. Jard. Bot. Natl. Belg. 39: 356 (1969). Type. Gabon: between Moubighou and Nzoundou, *Le Testu 6001*.

###### Note.

A replacement name, *Garciniangouniensis*, is created here for *Allanblackiagabonensis*, because the epithet *gabonensis* was used previously in *Garcinia* for a different species. The epithet *ngouniensis* is in reference to Gabon’s Ngounié province, an area where many specimens of this species have been collected.

##### ﻿ Garcinia oleosperma

Taxon classificationPlantaeMalpighialesClusiaceae

P.W.Sweeney nom. nov.

DD056EBC-054B-56AC-9211-F4280BD9BAA2

urn:lsid:ipni.org:names:77338406-1

Allanblackia floribunda Oliv., J. Linn. Soc., Bot. 10: 43 (1867). Type. Cameroon: Cameroon River, *Mann 2193*.

###### Note.

A replacement name, *Garciniaoleosperma*, is created here for the type species (*A.floribunda*) of the genus *Allanblackia*, because the epithet *floribunda* was used previously in *Garcinia* for a different species. The epithet *oleosperma* is in reference to the seeds that have a high oil content and are an important source of vegetable oil in tropical western Africa (Crockett 2015).

##### ﻿ Garcinia staneriana

Taxon classificationPlantaeMalpighialesClusiaceae

(Exell & Mendonça) P.W.Sweeney comb. nov.

FFC546E9-C7B8-5D42-A3E1-300C3D72FA02

urn:lsid:ipni.org:names:77338407-1

Allanblackia staneriana Exell & Mendonça, J. Bot. 74(Suppl.): 20 (1936). Type. Angola: Belize, *Grossweiler 8221*.

##### ﻿ Garcinia stuhlmannii

Taxon classificationPlantaeMalpighialesClusiaceae

(Engl.) P.W.Sweeney comb. nov.

5BC5AF3D-71B6-5C5A-A37B-3C77C900D070

urn:lsid:ipni.org:names:77338408-1

Allanblackia stuhlmannii (Engl.) Engl., H.G.A.Engler & K.A.E.Prantl, Nat. Pflanzenfam., Nachtr. 1: 249 (1897). Type. Tanzania: Usambara, *Holst 2296*.

##### ﻿ Garcinia ulugurensis

Taxon classificationPlantaeMalpighialesClusiaceae

(Engl.) P.W.Sweeney comb. nov.

1158C3EB-CEB6-56EB-BDB3-3C529EB11A58

urn:lsid:ipni.org:names:77338409-1

Allanblackia ulugurensis Engl., Bot. Jahrb. Syst. 28: 435 (1900). Type. Tanzania: Sudost Uluguru, *Stuhlmann 8773*.

#### *Garcinia* Lineage

***Leaves*** with druse crystals in the mesophyll (﻿Vesque 1889, 1893). ***Flowers*** without nectariferous antesepalous appendages or intrastaminal rings and disks (Sweeney 2010). Staminate flowers in many sections with pistillodes (but usually absent in sections *Brindonia*, *Hebradendron*, and *Macrostigma*) and anthers of various shapes.

The *Garcinia* lineage contains eight sections as circumscribed below and corresponds to Lineage B in Sweeney (2008).

##### ﻿4. Garcinia section Paragarcinia

Taxon classificationPlantaeMalpighialesClusiaceae

(Baillon) Vesque, Monogr. Phan. [A. DC. & C. DC.] 8: 254 (1893). Clade 3

75AD4154-BB84-5E41-91F2-A1FB55F064D5

Figs 2 , 3

###### Type.

*Ochrocarposdecipiens* Baill., Adansonia 11: 370 (1876) [≡*Garciniadecipiens* (Baill.) Vesque, Monogr. Phan. [A.DC. & C.DC.] 8: 482 (1893)].

###### Distinguishing sectional characters.

***Flowers*** with two (usually) sepals, fused in bud. Staminate flowers with a pistillode, stamens arranged into four (up to eight) fascicles with sessile to subsessile, two-thecous anthers. Ovaries four locular, stigmas weakly lobed. ***Fruits*** with smooth walls. ***Inflorescences*** terminal or axillary with few to many flowers. Afrotropics (Madagascar and Comoros).

This section contains the *Garcinia* species that were formerly placed into the segregate genus *Ochrocarpos*. The twelve species in this section are endemic to Madagascar and Comoros (Sweeney and Rogers 2008).

###### Species.

*Garciniacerasifer* (H.Perrier) P.F.Stevens; *G.dauphinensis* P.W.Sweeney & Z.S.Rogers; *G.decipiens* Vesque; *G.evonymoides* (Planch. & Triana) P.W.Sweeney & Z.S.Rogers; *G.goudotiana* (Planch. & Triana) P.W.Sweeney & Z.S.Rogers; *G.madagascariensis* (Planch. & Triana) Pierre; *G.multifida* (H. Perrier) P.W.Sweeney & Z.S.Rogers; *G.orthoclada* Baker; *G.parvula* (H. Perrier) P.W.Sweeney & Z.S.Rogers; *G.pauciflora* Baker; *G.tsaratananensis* (H. Perrier) P.W.Sweeney & Z.S.Rogers.

##### ﻿5. Garcinia section Discostigma

Taxon classificationPlantaeMalpighialesClusiaceae

(Haask.) Hook.f., Gen. Pl. [Benth. & Hook.f.] 1: 174 (1862). Clade 4

E3F1601A-5208-5A4E-A59B-77E44F4C632B

Figs 2 , 3

###### Basionym.

*Discostigma* Hassk., Flora 25(2, Beibl.): 33 (1842).

###### Type.

*Discostigmarostratum* Hassk., Flora 25(2, Beibl.): 33 (1842) [≡*Garciniarostrata* (Hassk.) Miq., Ann. Mus. Bot. Lugduno-Batavi 1(7): 209 (1864)].

###### Distinguishing sectional characters.

***Flowers*** with four sepals and petals. Staminate flowers with a pistillode, stamens arranged into four fascicles that are distally covered with sessile to subsessile, two-thecous anthers. Ovaries bilocular (or unilocular; four-locular in *G.yunnanensis*), stigmas unlobed and smooth. ***Fruits*** with a smooth surface and capped with a conspicuous discoid stigma, sepals caducous in fruits. ***Inflorescences*** terminal or axillary with few to many flowers. Indomalaya, tropical Australasia, and Oceania.

Sweeney (2008) noted that there were two groups of species placed into Discotigma by Jones (1980) that differed from typical members of the section by their androecial morphology. One group of species differs by having their stamens fused to the petals and includes *G.balansae*, *G.lanessanii* Pierre, *G.terpnophylla* Thwaites, and *G.warrenii* F.Muell. The position of *G.warrenii* in the trees presented here and in Sweeney (2008) suggests that some of these species may be better placed within Macrostigma (clade 9); however, our molecular analyses find strong support for placement of *G.balansae* within *Discostigma*. The second group of species is restricted to New Guinea, the Philippines, and Taiwan and includes *G.dives* Pierre, *G.hunsteinii* Lauterb., *G.linii* C. E. Chang, *G.luzoniensis* Merrill, and *G.palawanensis* Elmer (Jones 1980). This latter group is reported to have peltate anthers, like species of section Hebradendron (sensu Jones 1980); however, Jones (1980) placed them into Discostigma because they share the same stamen arrangement and pollen apertures as typical members of the section. Species representing the *G.dives* group have not yet been included in molecular phylogenetic analyses. *Garciniaanomala* was placed into Section Garcinia by Jones (1980), but excluded from that section by Nazre et al. (2018), due to its possession of axillary inflorescences in thyrses and stamens being united into an unlobed annular mass. Fruit characters suggest that this species belongs to Section Discostigma; however, the stamens are arranged into a ring.

In our ITS phylogeny, two species not treated by Jones (1980), *G.archboldiana* A.C. Sm. and *G.engleriana* A.C.Sm., are weakly supported as sister to Discostigma; however, in the chloroplast phylogeny these two species are shown as sister to a larger clade comprised of sections *Brindonia*, *Garcinia*, *Hebradendron*, and *Macrostigma*. The staminate flowers of *G.archboldiana* and *G.engleriana* lack pistillodes and they have deeply branched fascicles with numerous subpeltate anthers (Smith 1941). We leave these species unplaced. Future molecular and morphological work may warrant the placement of these species into their own section.

###### Species.

*Garciniaapetala* Pierre; *G.balansae* Pierre; *G.balica* Miq.; *G.binnendijkii* Pierre; *G.boerlagii* Pierre; *G.brevirostris* Scheff.; *G.cadelliana* King; *G.calophylla* Pierre; *G.calophyllifolia* Ridl.; *G.caudiculata* Ridl.; *G.cordata* Merr.; *G.cuneifolia* Pierre; *G.cuspidata* King; *G.diversifolia* King; *G.dives* Pierre; *G.dryobalanoides* Pierre; *G.enthaematoeides* Lauterb.; *G.gitingensis* Elmer; *G.grandifolia* (Choisy) Pierre; *G.hasskarlii* Pierre; *G.havilandii* Stapf; *G.holttumii* Ridl.; *G.hunsteinii* Lauterb.; *G.jensenii* W.E.Cooper; *G.keenania* Pierre; *G.kwangsiensis* Merr. ex F.N.Wei; *G.lanceola* Ridl.; *G.lancilimba* C.Y.Wu ex Y.H.Li; *G.lanessanii* Pierre; *G.linearis* Pierre; *G.linii* C.E. Chang; *G.luzoniensis* Merr.; *G.memecyloides* Ridl.; *G.merguensis* Wight; *G.microphylla* Merr.; *G.minimiflora* Ridl.; *G.minutiflora* Ridl.; *G.monantha* Ridl.; *G.multiflora* Champ. ex Benth.; *G.murtonii* Whitmore; *G.myrtifolia* A.C.Sm.; *G.novoguineensis* Vesque; *G.picrorhiza* Miq.; *G.rostrata* (Hassk.) Miq.; *G.salakensis* Pierre; *G.sampitana* Diels; *G.santisukiana* Ngerns. & Suddee; *G.sarawhensis* Pierre; *G.scaphopetala* B.L.Burtt; *G.tauensis* Lauterb.; *G.terpnophylla* Thwaites; *G.tetralata* C.Y.Wu ex Y.H.Li; *G.travancorica* Bedd.; *G.treubii* Pierre; *G.umbonata* Lauterb.; *G.versteegii* Lauterb.; *G.vitiensis* (A. Gray) Seem.; *G.wollastonii* Ridl.; *G.zichii* W.E.Cooper.

##### ﻿6. Garcinia section Brindonia

Taxon classificationPlantaeMalpighialesClusiaceae

(Thouars) Choisy, Mém. Soc. Hist. Nat. Paris 1: 230 (1823). Clade 5

AC7FA13D-3157-5279-8E17-F86A8AA84363

Figs 2 , 3

###### Basionym.

*Brindonia* Thouars, Dict. Sci. Nat. [F. Cuvier] 5: 339 (1806).

###### Type.

*Brindoniaoxycarpa* Thouars, Hist. Veg. Isles Austr. Afr. Ed. 2 t. 27 (1805) [≡*Garciniaoxycarpa* (Thouars) P.W.Sweeney, comb. nov.; *Garciniaindica* (Thours) Choisy Mém. syn. nov.]. The copy of *Histoire des végétaux recueillis dans les isles australes d’Afrique* ed. 2 at Kew bears the date 1805 (Baker 1894) and contains six plates (25–30) that do not have accompanying text in the main body of the publication (see also Hiern 1900). Plate 27 is labeled “*Brindoniaoxycarpa*” and it depicts two flowering branches, fruit, and dissected flowers (pers. obs). This suffices as an illustration with analysis and thus *Brindoniaoxycarpa* is validly published as per Articles 38.1, 38.7, and 38.8 of the ICN (Turland et al. 2018). Some (e.g. Hiern 1900) have considered *B.oxycarpa* a synonym of *Garciniaindica* (Thours) Choisy Mém. Soc. Hist. Nat. Paris 1: 230 (1823) [≡*Brindoniaindica* Thouars, Dict. Sci. Nat. [F. Cuvier] 5: 340 (1806)]; however, if these two taxa are considered synonymous, the epithet *oxcycarpa* would have priority.

###### Distinguishing sectional characters.

***Flowers*** with four sepals and petals. Staminate flowers without a pistillode (usually), stamens united into a single central bundle (or ring when pistillode present), anthers four-thecous (but in some species two-thecous). Ovaries multilocular, stigmas divided into distinct rays and usually papillate. ***Fruits*** in many species with furrows or grooves along the septal radii. ***Inflorescence***s terminal or axillary with one to many flowers. Afrotropics (Madagascar), Indomalaya, tropical Australasia, and Oceania.

Three species treated as Garcinia by Jones (1980) (i.e., *G.costata* Hemsl. ex King, *G.maingayi* Hook.f., and *G.trianii* Pierre) form a clade sister to clade 5, the latter largely corresponding to Brindona sensu Jones (1980). While they share some features (e.g. tendency to have furrowed/grooved (very shallow in *G.maingayi* and *G.trianii*), multilocular fruits) with *Brindonia* sensu Jones (1980), they have other features (i.e., pistillodes, stamens arranged into a ring, and two-thecous anthers) that are not typical of the section. While it would be tempting to recognize a separate section for these species, pistillodes and stamens arranged into a ring are also shared by *G.atroviridis* Griff. ex T.Anderson and *G.pedunculata* Roxb. ex Buch.-Ham., two species that were included in Brindonia by Jones (1980). It is noteworthy that *G.atroviridis* is the first branching lineage within clade 5, which together with the *G.costata*/*G.maingayi*/*G.trianii* clade form a basal grade within Brindonia (as circumscribed here).

*Garcinia* usually has an indehiscent drupe or berry (Stevens 2007). The genus *Clusianthemum* Vieill. was established by Vieillard for a new Caledonian species having a capsular fruit (*C.pedicellatum* Vieill.). Later, another capsular genus, *Septogarcinia* was established by Kostermans (1962) for *S.sumbawaensis* Kosterm., from Sumbawa (Indonesia), obviously not knowing about Vieillard’s *Clusianthemum*. Notably, several species of NC*Garcinia*, viz. *G.virgata* Vieill. ex Guillaumin, *G.neglecta* Vieill. and *G.comptonii* Baker f. have capsular fruits (Fig. 1). Jones (1980) did not mention *Clusianthemum* in her treatment but included *Septogarcinia* in Garciniasect.Brindonia. Jones (1980) does not cite any NC capsular species. The newly described *G.urceolata* is also suspected of having dehiscent fruits (Munzinger et al. 2021). All these species are morphologically similar and could result from *in situ* (within NC) diversification, and all sampled species with dehiscent fruit are found in a strongly supported subclade within clade 5. We do not have sequence material of *G.septogarcinia* I.M. Turner & L.V.S. Jenn. to determine whether that character is an autapomorphy of a dehiscent fruit clade, or if it evolved at least twice, in New Caledonia and Sumbawa. Staminate floral morphology supports placement of *G.septogarcinia* I.M. Turner & L.V.S. Jenn. into Brindona (Medellín-Zabala and Marinho 2015).

###### Species.

*Garciniaadinantha* A.C.Sm. & S.P.Darwin; *G.amabilis* Kaneh. & Hatus.; *G.amboinensis* Spreng.; *G.angustifolia* A.C. Sm.; *G.assamica* J.Sarma, Shameer & N.Mohanan; *G.assugu* Lauterb.; *G.asterandra* Jum. & H.Perrier; *G.atroviridis* Griff. ex T.Anderson; *G.balimensis* A.C. Sm.; *G.bancana* Miq.; *G.beccarii* Pierre; *G.bicolorata* Elmer; *G.binucao* (Blanco) Choisy; *G.borneensis* Pierre; *G.chapelieri* (Planch. & Triana) H.Perrier; *G.cochinchinensis* (Lour.) Choisy; *G.comptonii* Baker f.; *G.conicarpa* Wight; *G.corallina* Vieill.; *G.costata* Hemsl. ex King; *G.cowa* Roxb. ex DC.; *G.crassiflora* Jum. & H.Perrier; *G.dallmannensis* Kaneh. & Hatus.; *G.delpyana* Pierre; *G.dhanikhariensis* S.K.Srivast.; *G.dioica* Blume; *G.emarginata* Lauterb.; *G.erythrosepala* Y.H.Li; *G.esculenta* Y.H.Li; *G.fruticosa* Lauterb.; *G.fusca* Pierre; *G.griffithii* T.Anderson; *G.gummi-gutta* (L.) N.Robson; *G.horsfieldiana* Pierre; *G.hygrophila* Lauterb.; *G.indica* (Thouars) Choisy; *G.klinkii* Lauterb.; *G.korthalsii* Pierre; *G.lanceifolia* Roxb.; *G.lauterbachiana* A.C.Sm.; *G.ledermannii* Lauterb.; *G.leggeae* W.E.Cooper; *G.loheri* Merr.; *G.macgregorii* Merr.; *G.macrantha* A.C.Sm.; *G.maingayi* Hook. f.; *G.maluensis* Lauterb.; *G.mestonii* F.M.Bailey; *G.microstigma* Kurz; *G.minahassensis* Pierre; *G.miquelii* Pierre; *G.myristicifolia* Pierre; *G.nigrolineata* Planch. ex T.Anderson; *G.oblongifolia* Champ. ex Benth.; *G.oligophlebia* Merr.; *G.oliveri* Pierre; *G.oreophila* Lauterb.; *G.oxycarpa* (Thours) P.W.Sweeney; *G.pachyantha* A.C.Sm.; *G.pachypetala* Lauterb.; *G.pallida* Lauterb.; *G.parvifolia* (Miq.) Miq.; *G.pedunculata* Roxb. ex Buch.-Ham.; *G.ponapensis* Lauterb.; *G.quaesita* Pierre; *G.ramosii* Merr.; *G.riparia* A.C.Sm.; *G.rubra* Merr.; *G.rubriflora* Boerl.; *G.sabangensis* Lauterb.; *G.samarensis* Merr.; *G.schomburgkiana* Pierre; *G.segmentata* Kosterm.; *G.septogarcinia* I.M.Turner & L.V.S.Jenn.; *G.siripatanadilokii* Ngerns., Meeprom, Boonth., Chamch. & Sinbumr.; *G.solomonensis* A.C.Sm.; *G.sopsopia* (Buch.-Ham.) Mabb.; *G.stigmacantha* Pierre; *G.succifolia* Kurz; *G.sulphurea* Elmer; *G.tetrandra* Pierre; *G.teysmanniana* Scheff.; *G.trianii* Pierre; *G.urceolata* Munzinger, Bruy & M.Pignal; *G.valetoniana* Lauterb.; *G.vidua* Ridl.; *G.virgata* Vieill. ex Guillaumin; *G.viridiflora* Ridl.; *G.wallichii* Choisy; *G.xishuanbannaensis* Y.H.Li; *G.zeylanica* Roxb.

##### ﻿7. Garcinia L. section Garcinia

Taxon classificationPlantaeMalpighialesClusiaceae

. Clade 6

0C42771E-76DF-5E1D-8F69-311EBFF1DB89

Figs 2 , 3

###### Type.

*Garciniamangostana* L., Sp. Pl. 1: 443 (1753).

###### Distinguishing sectional characters.

***Flowers*** with four sepals and four petals. Staminate flowers often with a pistillode, stamens united into a single four-lobed or four-angled bundle, anthers two-thecous. Ovaries multilocular and stigmas with or without lobes and smooth or corrugated. ***Fruits*** with a smooth surface. ***Inflorescences*** terminal and comprised of simple cymes (Nazre et al. 2018). Indomalaya and tropical Australasia.

This section was recently monographed by Nazre et al. (2018) who recognized 13 species in the section and noted that species in the section share terminal inflorescences of simple cymes, stamens united into a single four-lobed or four-angled bundle, and fruits with a smooth surface. Based on morphological and molecular data he excluded several species that were included in this section by Jones (1980); our molecular results fully support his decisions (see discussion under clade 5).

###### Species.

*Garciniaacuticosta* Nazre; *G.celebica* L.; *G.diospyrifolia* Pierre; *G.discoidea* Nazre; *G.exigua* Nazre; *G.harmandii* Pierre; *G.mangostana* L.; *G.mangostifera* Kaneh. & Hatus.; *G.nitida* Pierre; *G.ochracea* Nazre; *G.penangiana* Pierre; *G.rigida* Miq.; *G.sangudsangud* Nazre; *G.sibeswarii* Shameer, J.Sarma, N.Mohanan & A.Begum; *G.venulosa* (Blanco) Choisy.

##### ﻿8. Garcinia section Hebradendron

Taxon classificationPlantaeMalpighialesClusiaceae

Planch. & Triana, Ann. Sci. Nat., Bot. sér. 4, 14: 349 (1860). Clade 7

C48EADD1-78DC-5593-A97B-C04FF60736F7

Figs 2 , 3

###### Basionym.

*Hebradendron* Graham, Companion Bot. Mag. 2: 199 (1837), *nom. illeg. superfl.* The genus name *Hebradendron* is illegitimate (superfluous as per Article 52.1, Turland et al. 2018) because Graham (1837) included within it *Stalagmitiscambogioides* Murray, Commentat. Soc. Regiae Sci. Gott. ix. 1787-88 (1789) 173 [≡*Hebradendroncambogioides* (Murray) Graham, Companion Bot. Mag. 2: 199, t. 27 (1837)], the type of *Stalagmitis* Murray, Commentat. Soc. Regiae Sci. Gott. 9: 173 (1789). Later, Planchon and Triana (1860) published GarciniasectionHebradendron Planch. & Triana, Ann. Sci. Nat., Bot. sér. 4, 14: 349 (1860), which according to Article 58.1 (Turland et al. 2018) can be considered a replacement name.

###### Type.

*Stalagmitiscambogioides* Murray, Commentat. Soc. Regiae Sci. Gott. 9: 173 (1789) [≡*Garciniacambogioides* (Murray) Headland, Man. Mater. Med. Therap. [Royle], ed. 3. 339 (1856); ≡*Hebradendroncambogioides* (Murray) Graham, Companion Bot. Mag. 2: 199, t. 27 (1837)]. See Shameer and Mohanan (2020) for a discussion about the priority of *G.cambogioides* (Murray) Headland over *G.morella* (Gaertn.) Desr.

###### Distinguishing sectional characters.

***Flowers*** sessile to subsessile and with four sepals and four petals. Staminate flowers without a pistillode, stamens united into a single central bundle, anthers unilocular and peltate with circumscissile dehiscence or with multiple chambers that dehisce via pores. Ovaries multilocular, stigmas lobed and variously ornamented, often papillate. ***Fruits*** with smooth surface, pedicels thick in fruit. ***Inflorescences*** axillary with one to a few flowers. Indomalaya and tropical Australasia.

###### Species.

*Garciniaacuminata* Planch. & Triana; *G.blumei* Pierre; *G.bonii* Pit.; *G.burkillii* Whitmore; *G.calycina* Kurz; G.*cambogioides* (Murray) Headland; *G.cantleyana* Whitmore; *G.choisyiana* (Choisy) Wall. ex Planch. & Triana; *G.daedalanthera* Pierre; *G.desrousseauxii* Pierre; *G.dumosa* King; *G.fuscopetiolata* Lauterb.; *G.garciae* Elmer; *G.gaudichaudii* Planch. & Triana; *G.gjellerupii* Lauterb.; *G.grahamii* Pierre; *G.hanburyi* Hook.f.; *G.hendersoniana* Whitmore; *G.heterandra* Wall. ex Planch. & Triana; *G.hopii* H.Toyama & V.S.Dang; *G.idenburgensis* A.C.Sm.; *G.imberti* Bourd.; *G.jaweri* Lauterb.; *G.lateriflora* Blume; *G.microcarpa* Pierre; *G.microtropidiiformis* Kaneh. & Hatus.; *G.mindanaensis* Merr.; *G.murdochii* Ridl.; *G.oligantha* Merr.; *G.poilanei* Gagnep.; *G.pullei* Lauterb.; *G.rheedei* Pierre; *G.schlechteri* Lauterb.; *G.scortechinii* King; *G.subtilinervis* F.Muell.; *G.timorensis* Zipp. ex Span.; *G.uniflora* King; *G.urophylla* Scort. ex King; *G.wightii* T.Anderson.

##### ﻿9. Garcinia section Tagmanthera

Taxon classificationPlantaeMalpighialesClusiaceae

Pierre, Fl. Forest. Cochinch. Vol. 1, Fasc. 6, 17 (1883). Clade 8

FF3E6C00-46A9-5979-A90E-DD86C219D5A8

Figs 2 , 3

###### Type.

*Garciniapunctata* Oliv., Fl. Trop. Afr. 1: 167 (1868).

###### Distinguishing sectional characters.

***Staminate flowers*** with a pistillode, stamens arranged into four (rarely two) strap-shaped fascicles each with a single row of sessile, recurved, and sometimes multilocellate anthers at the end. Ovaries four locular, stigmas weakly lobed. ***Fruits*** with smooth surface. ***Inflorescences*** terminal or axillary with one to a few flowers. Afrotropics.

###### Species.

*Garciniaacutifolia* N.Robson; *G.afzelii* Engl.; *G.bifasciculata* N.Robson; *G.buchananii* Baker; *G.buchneri* Engl.; *G.elliotii* Engl.; *G.epunctata* Stapf; *G.huillensis* Welw. ex Oliv.; *G.lujae* de Wild.; *G.mannii* Oliv.; *G.preussii* Engl.; *G.punctata* Oliv.; *G.tanzaniensis* Verdc.

##### ﻿10. Garcinia section Macrostigma

Taxon classificationPlantaeMalpighialesClusiaceae

Pierre, Fl. Forest. Cochinch. Vol. 1, Fasc. 6, 36 (1883). Clade 9

8A6A60F7-7D5E-5203-825D-DA04C37A7C36

Figs 2 , 3

###### Type.

*Garcinialatissima* Miq., Ann. Mus. Bot. Lugduno-Batavi 1: 209 (1864).

###### Distinguishing sectional characters.

***Staminate flowers*** lacking pistillode (usually, but rudimentary or well-developed pistillode present in some species), stamens united into central column (sometimes lobed with lobes equaling number of petals) or into completely separate antepetalous fascicles, androecium often adnate to the petals to varying degrees, anthers two-thecous. Ovaries four (three) to eight locular, stigmas unlobed and smooth or divided and papillose. ***Fruits*** with smooth walls or faintly to deeply furrowed/grooved. ***Inflorescences*** axillary or terminal with one to many flowers. Indomalaya, tropical Australasia, and Oceania.

This section includes chiefly species that were included in Jones’ (1980) sections *Macrostigma*, *Mungotia*, and *Tripetalum*. This is perhaps the most heterogenous of the sections recognized here and it is difficult to point to a single character shared by all of the species in the section. Many species, especially those that were placed into sections *Macrostigma* and *Tripetalum*, often have stamen bundles adnate to the petals. It has been suggested that highly branched, anastomosing exudate-containing canals on the adaxial leaf surface may be a synapomorphy for this clade (Sweeney 2008); however, this has not been comprehensively studied across the genus and may not be a reliable character for determining sectional placement (see Cooper 2013). Many species possess leaves with elliptic, elliptic-obovate, or obovate leaves with thin, closely spaced (ca. <5 mm) secondary veins. Other possible features uniting species in the group include the presence of an exotegmen and non-garcinioid type seed germination (see Brandza 1908; Stevens 2007). Further study is needed to confirm the distribution/presence of these characters.

In the phylogeny, this clade includes three species that have been variously placed into other sections by other authors (Lauterbach 1922; Jones 1980): *G.hollrungii*, *G.prainiana*, and *G.warrenii*. In addition to molecular data, these species have morphology that supports their placement into Macrostigma.

###### Species.

*Garciniaamplexicaulis* Vieill. ex Pierre; *G.branderhorstii* Lauterb.; *G.brassii* C.T.White; *G.carolinensis* (Lauterb.) Kosterm.; *G.crassifolia* Seeth.; *G.crassinervis* (Warb.) Kosterm.; *G.cymosa* (K.Schum.) I.M.Turner & P.F.Stevens; *G.densiflora* Pierre; *G.gibbsiae* S.Moore; *G.hollrungii* Lauterb.; *G.latissima* Miq.; *G.moselleyana* Pierre; *G.multibracteolata* Merr.; *G.mungotia* Planch. ex Pierre; *G.nuntasaenii* Ngerns. & Suddee; *G.pachycarpa* (A.C.Sm.) Kosterm.; *G.pancheri* Pierre; *G.pedicellata* (G.Forst.) Seem.; *G.phuongmaiensis* V.S.Dang, H.Toyama & D.L.A.Tuan; *G.platyphylla* A.C.Sm.; *G.prainiana* King; *G.pseudoguttifera* Seem.; *G.puat* (Montrouz.) Guillaumin; *G.quadrilocularis* Seeth.; *G.russellii* W.E.Cooper; *G.sessilis* (G.Forst.) Seem.; *G.smithii* Kosterm.; *G.vieillardii* Pierre; *G.warrenii* F.Muell.

##### ﻿11. Garcinia section Dicrananthera

Taxon classificationPlantaeMalpighialesClusiaceae

Pierre, Fl. Forest. Cochinch. 1, Fasc. 6, 8 (1883).

AC4034A3-1B0F-5F8E-A3DC-1DFD3EE7C97C

###### Type.

*Garciniathorelii* Pierre, Fl. Forest. Cochinch. t. 62.

###### Distinguishing sectional characters.

***Leaves*** with prominent stipuliform structures. Staminate flowers with a pistillode, stamens united into an annular mass encircling and attached to the pistillode, anthers two-thecous. Ovaries one to two locular, stigmas unlobed and smooth. ***Fruits*** with smooth walls. ***Inflorescences*** axillary or terminal with three to many flowers. Indomalaya.

*Garcinianujiangensis* C.Y.Wu & Y.H.Li occupies an isolated position in the phylogeny, in a polytomy with clades 4 and 9. We resurrect Pierre’s Dicrananthera for a morphologically coherent group of species that was designated the “*Garciniastipulata*” group in Sweeney et al. (2022). Jones (1980) treated this group, using Pierre’s sectional name, as a subsection of *Discostigma* (GarciniasectionDiscostigmasubsectionDicrananthera (Pierre) S.W.Jones *nom. inval.* Art. 30.9, Turland et al. 2018). Species in this group collectively range from eastern India and Bhutan, east to southwest China, and south to Myanmar and Laos. In addition to *G.nujiangensis*, the section contains *G.yaatapsap* K. Armstr. & P.W. Sweeney, *G.paucinervis* Chun & F.C.How (1956: 12), *G.stipulata* T.Anderson, and *G.thorelii*Pierre (1882: t. [plate] 62). These species all share prominent stipuliform structures (rare in Clusiaceae, Stevens 2007), leaves with prominent, widely spaced, curved secondary veins and percurrent tertiaries, staminate flowers with numerous stamens united into an annular mass encircling and attached to the pistillode (in *G.paucinervis* and *G.nujiangensis* the stamens are described as being in four bundles (Chun and How 1956; Li 1981)), and ellipsoid fruits with a discoid stigma and one to two seeds. Future molecular phylogenetic analyses will confirm if species of the ‘stipulata’ group are monophyletic and whether they will remain a distinct clade separate from clade 4/section Discostigma.

###### Species.

*Garcinianujiangensis* C.Y.Wu & Y.H.Li; *G.paucinervis* Chun & F.C.How; *G.stipulata* T.Anderson; *G.thorelii* Pierre; *G.yaatapsap* K.Armstr. & P.W.Sweeney.

#### Unplaced species

*Garciniaanomala* Planch. & Triana; *G.archboldiana* A.C.Sm.; *G.blancoi* Pierre; *G.bracteata* C.Y.Wu ex Y.H.Li; *G.busuangaensis* Merr.; *G.caloneura* Boerl.; *G.ceramica* Boerl.; *G.clusiifolia* Ridl.; *G.engleriana* A.C.Sm.; *G.erythrosperma* Lauterb.; *G.fagraeoides* A.Chev.; *G.graminea* Kosterm.; *G.ituman* Merr.; *G.jelinckii* Kurz; *G.klossii* Ridl.; *G.linearifolia* Elmer; *G.longipedicellata* Kosterm.; *G.lucens* Pierre; *G.mammeoides* Kosterm.; *G.matsudae* Kaneh.; *G.montana* Ridl.; *G.moszkowskii* Lauterb.; *G.moulmeinensis* Pierre ex Vesque; *G.nubigena* Lauterb.; *G.pacifica* Merr.; *G.pallide-sanguinea* Lauterb.; *G.plena* Craib; *G.propinqua* Craib; *G.qinzhouensis* Y.X.Liang & Z.M.Wu; *G.ramulosa* Lauterb.; *G.rhizophoroides* Elmer; *G.rumiyo* Kaneh.; *G.rupestris* Lauterb.; *G.schraderi* Lauterb.; *G.squamata* Lauterb.; *G.subfalcata* Y.H.Li & F.N.Wei; *G.torensis* Lauterb.; *G.tuberculata* Lauterb.; *G.whitfordii* Merr.; *G.wichmannii* Lauterb.

### ﻿Taxonomy of the NC*Garcinia* species

The phylogenetic framework estimated in this study does not support the distinction between *G.* sp. “JT814” and *G.balansae* within NC, nor recover four species with multiple accessions as monophyletic (viz. *G.pedicellata*, *G.puat*, *G.comptonii*, *G.neglecta*), but confirms the distinction between NC species and both *G.vitiensis* and *G.adinantha* found in Fiji. Therefore, all NC species should still be considered as endemics. Also, *G.balansae* (belonging to clade 4/Discostigma) appears more closely related to species from Fiji (*G.myrtifolia*, *G.vitiensis*), Australia (*G.jensenii*) and southeast Asia (*G.brevirostris*, *G.merguensis*, *G.rostrata*, *G.lancilimba*, *G.tetralata*) than to any other NC species.

The four species with capsular fruits (*G.comptonii*, *G.neglecta*, *G.urceolata* and *G.virgata*; retrieved in clade 5/Brindonia) cannot be distinguished based on the present molecular data, but they form two pairs of species based on morphology and ecology. *Garciniaurceolata* and *G.virgata* have small leaves and were confused for a long time but differ by their flowers (green urceolate versus yellowish cup-like corolla), leaves and fruits. Both occur in dense humid forest on non-ultramafic substrates, but *G.urceolata* grows at higher elevation and in wetter conditions than *G.virgata*. *G.comptonii* appears restricted to maquis or forest edges on ultramafic substrates, while *G.neglecta* is mostly a forest tree on ultramafic and non-ultramafic substrates.

In the other NC clade (included in clade 9/Macrostigma), *G.vieillardii* is restricted to the northeast dense humid forest on non-ultramafic soils, above 550 m a.s.l., while *G.densiflora* occurs in the same area and also on non-ultramafic substrates but at lower elevation. In addition, it is more a rupicolous species. The three other species can be found on both substrates (ultramafic and non-ultramafic). *Garciniapuat* is restricted to dense humid forest at low elevations, while *G.pedicellata* is a coastal (including small islands) to medium elevation tree, growing in drier conditions than the three previously cited species. Finally, *G.amplexicaulis* is the species with the widest ecological amplitude, growing in open maquis to closed humid forest, from low to 900 m a.s.l., throughout all the main island.

## ﻿Conclusions

This study offers a way forward on a revised infrageneric classification of the species-rich genus *Garcinia*, based on both evolutionary history (as informed by molecular phylogenies) and morphology. We recognize eleven sections within *Garcinia*, list representative species and document distinctive morphological features for each. This classification provides an evolutionary-based foundation for future, much needed monographic work within the genus. Although additional phylogenetic analyses are warranted, by including more species and increasing the phylogenetic resolution, our phylogenetic results are a major contribution to the understanding of the evolutionary history of the genus and they provide a starting point for more ecological and evolutionary investigations as well as conservation planning and taxonomic work. Future revisionary efforts focused on some of the more speciose sections/clades recognized here (*Brindonia*, *Discostigma*, and *Hebradendron*) will certainly result in many species being reduced to synonymy and some new species being described. This was the case with a recent revision of Section Garcinia (Nazre et al. 2018). And, as more detailed taxonomic work is done, some species section reassignments are inevitable as are the erection of new sections to accommodate newly discovered clades with distinct suites of morphological characters.

One area that is particularly attractive for future research concerns the biogeographic history of the genus. A more complete knowledge of the spatio-temporal history of *Garcinia* would allow for a better understanding of the events that lead to the present geographic distribution of the genus. This would contribute to a growing body of knowledge about the biogeography of pantropically distributed clades and would provide additional data for exploring hypotheses about intercontinental disjunctions (e.g. Clayton et al. 2009; Couvreur et al. 2011; Baker and Couvreur 2013; Ruhfel et al. 2016; Torke et al. 2022). At a smaller scale, studying the origin of the ten endemic NC*Garcinia* species would also be valuable. Species diversification probably occurred after colonization of the territory by long-distance colonization and recent studies on other plant groups showed that in addition to the closest and largest landmass that is Australia, diverse geographic origins could be revealed (e.g. Duangjai et al. 2009; Del Rio et al. 2017; Samuel et al. 2019).

## Supplementary Material

XML Treatment for Garcinia section Xanthochymus

XML Treatment for Garcinia section Rheedia

XML Treatment for Garcinia section Allanblackia

XML Treatment for Garcinia guineensis

XML Treatment for Garcinia kisonghi

XML Treatment for Garcinia kimbiliensis

XML Treatment for Garcinia marienii

XML Treatment for Garcinia ngouniensis

XML Treatment for Garcinia oleosperma

XML Treatment for Garcinia staneriana

XML Treatment for Garcinia stuhlmannii

XML Treatment for Garcinia ulugurensis

XML Treatment for Garcinia section Paragarcinia

XML Treatment for Garcinia section Discostigma

XML Treatment for Garcinia section Brindonia

XML Treatment for Garcinia L. section Garcinia

XML Treatment for Garcinia section Hebradendron

XML Treatment for Garcinia section Tagmanthera

XML Treatment for Garcinia section Macrostigma

XML Treatment for Garcinia section Dicrananthera

## ﻿Additional information

### Author ORCIDs

Patrick Sweeney https://orcid.org/0000-0003-1239-189X

Jérôme Munzinger https://orcid.org/0000-0001-5300-2702

### Data availability

All of the data that support the findings of this study are available in the main text or Supplementary Information.

## Supplementary materials

Supplementary material 1 List of taxa and accessions used in this study Data type xlsx Explanation note The origin of DNA sequences is indicated, together with information on the herbarium voucher and distribution area of the taxon. In cases where the accepted name that was used in this study differed from the name provided in the original study that generated a sequence or voucher, the original name is provided (under “Prior determination”) along with a reference for the taxonomic decision. Accessions that were newly sequenced in this study have boldface text. All accessions were included in the nuclear DNA phylogenetic analysis (except three samples, as indicated in the first column) and a subset was included in the chloroplast DNA phylogenetic analysis, as indicated in the corresponding column. https://binary.pensoft.net/file/1002039This dataset is made available under the Open Database License (http://opendatacommons.org/licenses/odbl/1.0/). The Open Database License (ODbL) is a license agreement intended to allow users to freely share, modify, and use this Dataset while maintaining this same freedom for others, provided that the original source and author(s) are credited.Myriam Gaudeul, Patrick Sweeney, Jérôme Munzinger

Supplementary material 2 Molecular phylogeny of *Garcinia* L. based on *psbM-trnD* and Bayesian inference Data type jpeg Explanation note Posterior probabilities (PP) are indicated at each node of the cladogram. Nodes were collapsed when PP < 0.50. Species names appear in colors depending on their native distribution areas: light green, Tropical Africa; dark green, Madagascar and Western Indian Ocean islands; grey, Southeast Asia; purple, Australia; orange, New Guinea; red, New Caledonia; dark blue, Southwest Pacific islands. Distribution information was taken from the Plants of the World Online website (POWO 2023; also see the table of vouchers). A few species occur in several regions, and the color of the main (largest) region was used. All accessions were newly sequenced in this study. https://binary.pensoft.net/file/1002040This dataset is made available under the Open Database License (http://opendatacommons.org/licenses/odbl/1.0/). The Open Database License (ODbL) is a license agreement intended to allow users to freely share, modify, and use this Dataset while maintaining this same freedom for others, provided that the original source and author(s) are credited.Myriam Gaudeul, Patrick Sweeney, Jérôme Munzinger

Supplementary material 3 Molecular phylogeny of *Garcinia* L. based on *trnQ-rps16* and Bayesian inference Data type jpeg Explanation note Posterior probabilities (PP) are indicated at each node of the cladogram. Nodes were collapsed when PP < 0.50. Species names appear in colors depending on their native distribution areas: light green, Tropical Africa; dark green, Madagascar and Western Indian Ocean islands; grey, Southeast Asia; purple, Australia; orange, New Guinea; red, New Caledonia; dark blue, Southwest Pacific islands. Distribution information was taken from the Plants of the World Online website (POWO 2023; also see the table of vouchers). A few species occur in several regions, and the color of the main (largest) region was used. All accessions were newly sequenced in this study. https://binary.pensoft.net/file/1002041This dataset is made available under the Open Database License (http://opendatacommons.org/licenses/odbl/1.0/). The Open Database License (ODbL) is a license agreement intended to allow users to freely share, modify, and use this Dataset while maintaining this same freedom for others, provided that the original source and author(s) are credited.Myriam Gaudeul, Patrick Sweeney, Jérôme Munzinger

Supplementary material 4 Molecular phylogeny of *Garcinia* L. based on *rps16-trnK* and Bayesian inference Data type jpeg Explanation note Posterior probabilities (PP) are indicated at each node of the cladogram. Nodes were collapsed when PP < 0.50. Species names appear in colors depending on their native distribution areas: light green, Tropical Africa; dark green, Madagascar and Western Indian Ocean islands; grey, Southeast Asia; purple, Australia; orange, New Guinea; red, New Caledonia; dark blue, Southwest Pacific islands. Distribution information was taken from the Plants of the World Online website (POWO 2023; also see the table of vouchers). A few species occur in several regions, and the color of the main (largest) region was used. All accessions were newly sequenced in this study. https://binary.pensoft.net/file/1002042This dataset is made available under the Open Database License (http://opendatacommons.org/licenses/odbl/1.0/). The Open Database License (ODbL) is a license agreement intended to allow users to freely share, modify, and use this Dataset while maintaining this same freedom for others, provided that the original source and author(s) are credited.Myriam Gaudeul, Patrick Sweeney, Jérôme Munzinger

Supplementary material 5 Molecular phylogeny of *Garcinia* L. based on a combined ITS and chloroplast DNA (*psbM-trnD, trnQ-rps16* and *rps16-trnK*) dataset and Bayesian inference Data type jpeg Explanation note Posterior probabilities (PP) are indicated at each node of the cladogram. Nodes were collapsed when PP < 0.50. Species names appear in colors depending on their native distribution areas: light green, Tropical Africa; dark green, Madagascar and Western Indian Ocean islands; grey, Southeast Asia; purple, Australia; orange, New Guinea; red, New Caledonia; dark blue, Southwest Pacific islands. Distribution information was taken from the Plants of the World Online website (POWO 2023; also see the table of vouchers). A few species occur in several regions, and the color of the main (largest) region was used. https://binary.pensoft.net/file/1002043This dataset is made available under the Open Database License (http://opendatacommons.org/licenses/odbl/1.0/). The Open Database License (ODbL) is a license agreement intended to allow users to freely share, modify, and use this Dataset while maintaining this same freedom for others, provided that the original source and author(s) are credited.Myriam Gaudeul, Patrick Sweeney, Jérôme Munzinger
