# Supplementary figures and images for: ﻿An updated infrageneric classification of the pantropical species-rich genus Garcinia L. (Clusiaceae) and some insights into the systematics of New Caledonian species, based on molecular and morphological evidence
Source: PhytoKeys. 2024 Mar 15;239:73–105. doi: 10.3897/phytokeys.239.112563 (PMC10960151; doi:10.3897/phytokeys.239.112563)

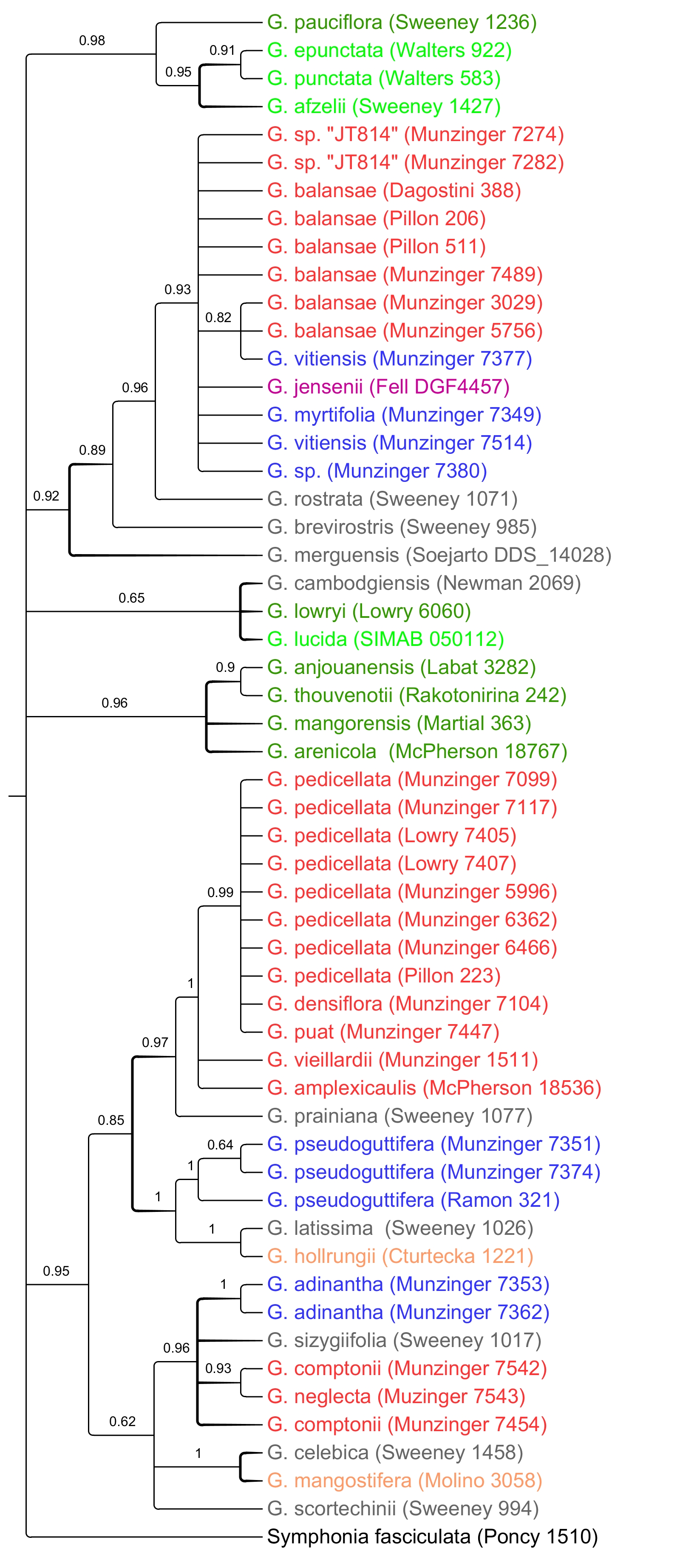

Supplement: Supplementary material 2 — Molecular phylogeny of Garcinia L. based on psbM-trnD and Bayesian inference [file phytokeys-239-073_article-112563__-s002.jpeg]

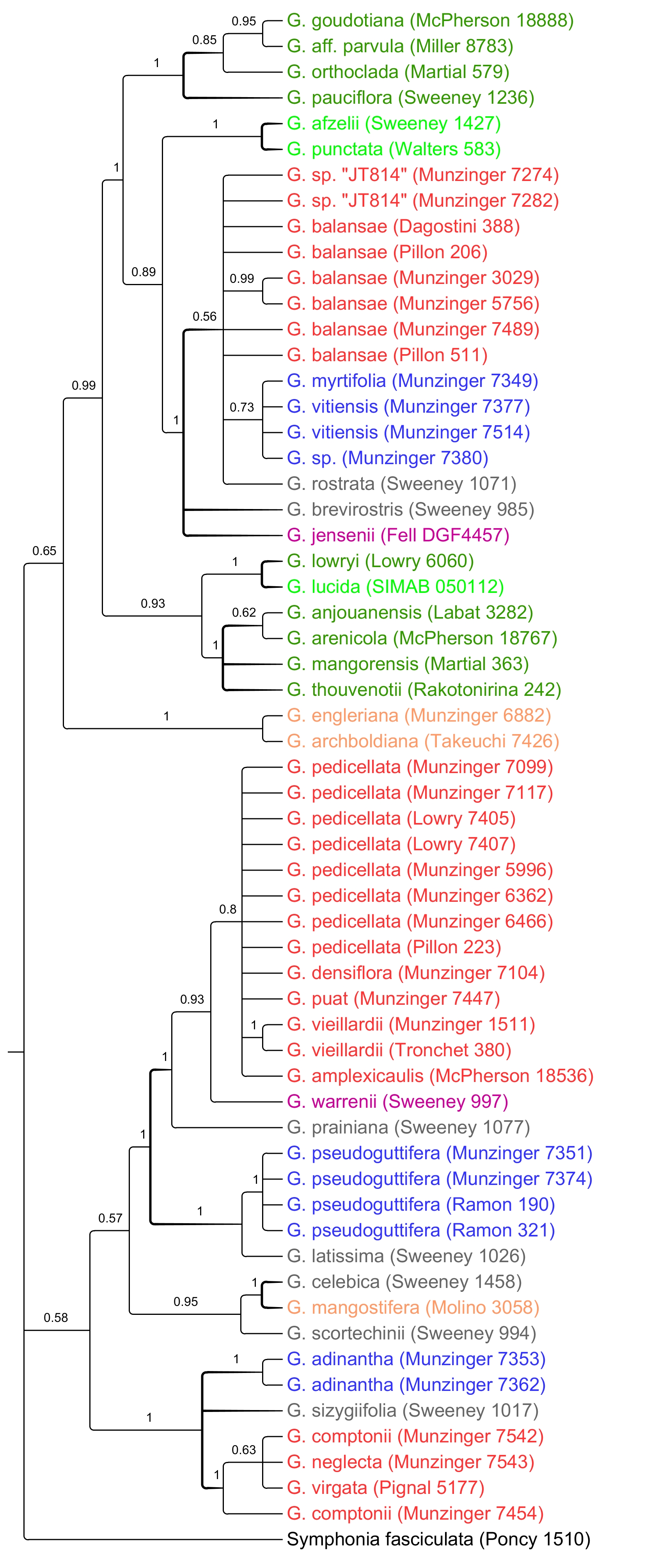

Supplement: Supplementary material 3 — Molecular phylogeny of Garcinia L. based on trnQ-rps16 and Bayesian inference [file phytokeys-239-073_article-112563__-s003.jpeg]

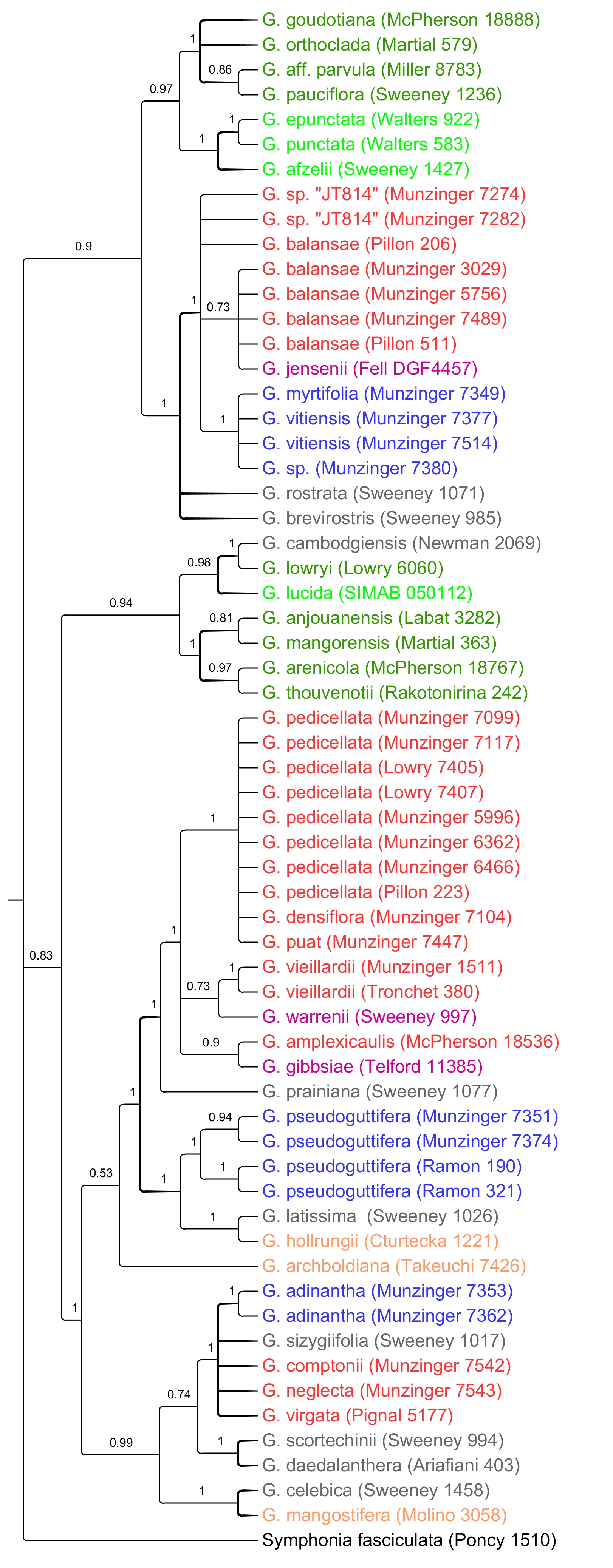

Supplement: Supplementary material 4 — Molecular phylogeny of Garcinia L. based on rps16-trnK and Bayesian inference [file phytokeys-239-073_article-112563__-s004.jpeg]

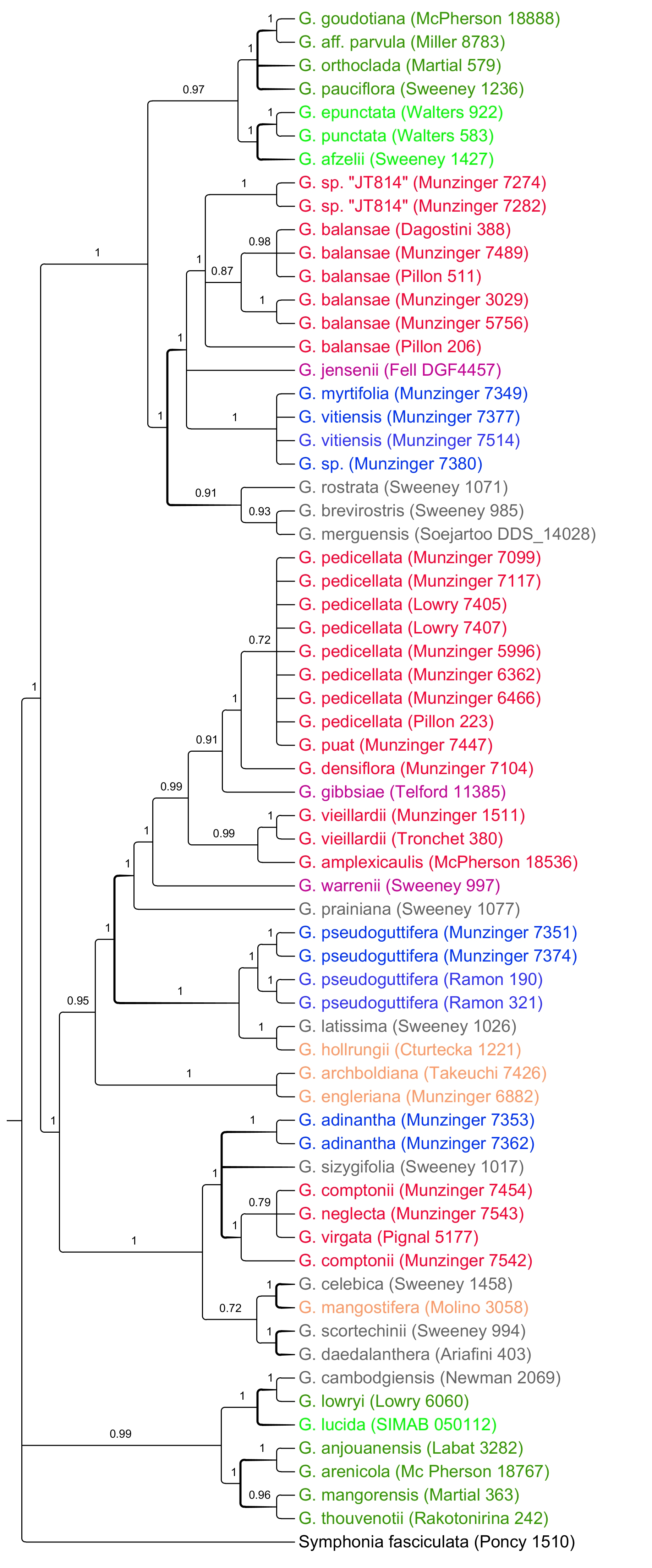

Supplement: Supplementary material 5 — Molecular phylogeny of Garcinia L. based on a combined ITS and chloroplast DNA (psbM-trnD, trnQ-rps16 and rps16-trnK) dataset and Bayesian inference [file phytokeys-239-073_article-112563__-s005.jpeg]
